# Coupled Excited-State Dynamics in N-Substituted 2-Methoxy-9-Acridones

**DOI:** 10.3389/fchem.2019.00129

**Published:** 2019-03-12

**Authors:** M. Carmen Gonzalez-Garcia, Pilar Herrero-Foncubierta, Silvia Castro, Sandra Resa, Jose M. Alvarez-Pez, Delia Miguel, Juan M. Cuerva, Emilio Garcia-Fernandez, Angel Orte

**Affiliations:** ^1^Departamento de Fisicoquimica, Facultad de Farmacia, Unidad de Excelencia en Quimica Aplicada a Biomedicina y Medioambiente (UEQ), Universidad de Granada, Granada, Spain; ^2^Departamento de Quimica Organica, Facultad de Ciencias, Unidad de Excelencia en Quimica Aplicada a Biomedicina y Medioambiente (UEQ), Universidad de Granada, Granada, Spain

**Keywords:** excited-state dynamics, fluorophores, excited-state proton transfer, excimers, kinetics, computational photophysics

## Abstract

Fluorophores of the acridone family have been widely employed in many applications, such as DNA sequencing, the detection of biomolecules, and the monitoring of enzymatic systems, as well as being the bases of intracellular sensors and even antitumoral agents. They have been widely used in fluorescence imaging due to their excellent photophysical properties, in terms of quantum yield and stability. However, frequently, the fluorescence emission data from acridones are not easily interpretable due to complex excited-state dynamics. The formation of π-stacking aggregates and excimers and excited-state proton transfer (ESPT) reactions usually result in emission features that are dependent on the experimental conditions. Therefore, an in-depth understanding of the dynamics involved in the excited-state transients of these dyes is mandatory for their appropriate application. Herein, we synthesized and fully characterized different 2-methoxy-9-acridone dyes. Their transient fluorescence emission spectra exhibited a complex dynamic behavior that can be linked to several excited-state reactions. We performed a thorough study of the excited-state dynamics of these dyes by means of time-resolved fluorimetry supported by computational calculations. All this allowed us to establish a multistate kinetic scheme, involving an ESPT reaction coupled to an excimer formation process. We have unraveled the rich dynamics behind this complex behavior, which provides a better understanding of the excited states of these dyes.

## Introduction

Fluorescence imaging can be a valuable tool for studying biomolecules in complex biological environments because of characteristics such as its great sensitivity, high spatial resolution, and ease to use (Nalbant et al., 2004). Nevertheless, the application of fluorescence intensity as a key parameter has some drawbacks since its use may require knowledge of the relative concentration of the fluorophore, and this is usually unknown in biological structures. Unlike other fluorescence parameters, the fluorescence lifetime is an intrinsic property of a fluorophore and therefore does not depend on the fluorophore concentration, as well as being independent of the excitation wavelength (Berezin and Achilefu, 2010). However, the fluorescence lifetime is highly sensitive to environmental factors, such as solvent polarity (Orte et al., 2016; Ripoll et al., 2018), conformational changes (Tomin, 2010), and excited-state reactions (Alvarez-Pez et al., 2001), among others. These characteristics together with the independence from the concentration of the fluorophore make fluorescence lifetime imaging (FLIM) an advantageous method over fluorescence intensity measurements. In addition, another advantage of using FLIM is that the fluorescence lifetime allows for discrimination between the fluorescence from different fluorophores, despite the overlap in their wavelengths of emission (Berezin and Achilefu, 2010). This property allows for differentiating between the autofluorescence of cells and tissues and that of the fluorescent labels that are used as probes. This discrimination is even more specific when the employed fluorescent probes have a long lifetime since the fluorophores that generate the biological background usually possess short lifetimes (Stevens et al., 2008; Berezin and Achilefu, 2010). Therefore, fluorophores with large lifetimes are of choice in biological sensing using FLIM (Ruedas-Rama et al., 2015). Indeed, by applying the FLIM methodology and the single molecule approach, we have been able to evaluate the transport of extracellular phosphate into preosteoblast cells during osteoblast differentiation (Paredes et al., 2013) and to monitor pH changes within the cellular cytoplasm using pH-sensitive nanoparticles (Orte et al., 2013).

Among the various groups of small fluorescent molecules with large lifetimes, those based on the acridone moiety show fluorescence emission in the 400–500 nm region with lifetimes >10 ns, as well as pH independence in the physiological range (Smith et al., 2004). Approximately 270 alkaloids of the acridone family have been described (Michael, 2017), and due to their multiple uses, a large number of acridone derivatives have also been synthesized. Among their most noteworthy properties are the effects of acridones and synthetic analogs as antineoplastic agents (Kuete et al., 2015; Fomani et al., 2016; Schelz et al., 2016). Antibacterial and antiviral activities have also been described (Wansi et al., 2006). Likewise, acridone derivatives have found diverse usage as detection reagents for biomolecules (Kitagawa et al., 1995) and metal ions (Fukuzumi and Ohkubo, 2002), as well as tunable photosensitizers for photoredox catalysis (Chen et al., 2018).

The acridone moiety presents a ketone in a tricyclic chromophoric system with planar geometry that favors π-stacking interactions between neighboring molecules, which may lead to the formation of excimers. An excimer is defined as a dimer between two identical monomers, one of them in the ground state and the other in the excited state (Lakowicz, 2006). The formation of an excimer is induced by the orientation of two chromophores, especially in the aggregated assemblies, upon absorption of a photon, which is associated with the π-stacking interactions. Excimers normally show redshifted absorption and emission spectra with respect to those of the monomer.

Aromatic molecules with unoccupied π^*^ molecular orbitals can also undergo excited-state intermolecular proton transfer, as mediated by solvent molecules. In some cases, proton transfer may occur in hydrogen-bonded dimers. The great majority of acridone derivatives present these π-stacking interactions as well as intermolecular hydrogen bond formation (Liu et al., 2000). Precisely, the formation/cleavage of the π-stacking interactions provide some crystals of acridone derivatives with molecular-packing-dependent emission properties, which are useful in photonic devices and biological sensing (Chen et al., 2016; Takeda and Akutagawa, 2016; Bricks et al., 2018). A complex, hydrogen bonded with the solvent, is formed in the ground state so that its absorption and emission spectra are also redshifted relative to those of the non-hydrogen-bonded solutes (Lakowicz, 2006). Conversely, electron acceptors with unoccupied π^*^ orbitals can accept electrons when the excited state is reached. In this case, the increased electronic density results in a decrease in the excited-state dissociation constant. The number of photobases that have been investigated is substantially lower than that of photoacids, limiting knowledge about the possible usefulness of photobases (Sheng et al., 2018).

Although a few reports on the fluorescence spectra and fluorescence lifetimes of some acridone derivatives in solution have been published (Smith et al., 2004), as well as their behavior as very weak acids in their excited states (Schulman and Sturgeon, 1977), a detailed investigation of their photophysics in solution has not yet been described. Therefore, it is essential to elucidate the dynamics of the excited-state in the molecular and supramolecular forms that are present at the different pH-values to thoroughly understand the photophysics of acridones in solution.

The dynamics of the excited-state processes of fluorescent molecules can be made accessible by time-resolved fluorescence measurements. Usually, the fluorescence decay trace of an excited-state system is described by a sum of exponential functions in terms of the decay times and their preexponential factors. However, the most important parameters, when an excited-state dynamic process is present, are the rate constants defining the excited states along with the excitation and emission spectra associated with the species involved in the kinetic system (Boens et al., 2004). To determine these parameters, a multidimensional fluorescence decay data surface should be measured under a variety of experimental conditions, and from the resulting set of fluorescence decays, the rate constants and the spectra associated with excitation and emission can be linked and determined. Such a global analysis, in which the rate constants of the excited-state processes and the associated spectral parameters are the linked variables to be calculated, is called global compartmental analysis (GCA) (Boens and Ameloot, 2006).

Herein, we prepared two different N-substituted 2-methoxy-9-acridone derivatives and studied their photophysical dynamics by formulating an appropriate kinetic scheme, involving all the detected excited-state reactions, and analyzed these species using global analysis. Intricate excited-state dynamics were found to involve coupled excited-state reactions, including proton transfer and excimer formation. To the best of our knowledge, this report is the first to resolve such coupled excited-state dynamics.

## Materials and Methods

### Syntheses of N-modified 2-Methoxy-9-Acridones

**Figure 1:**
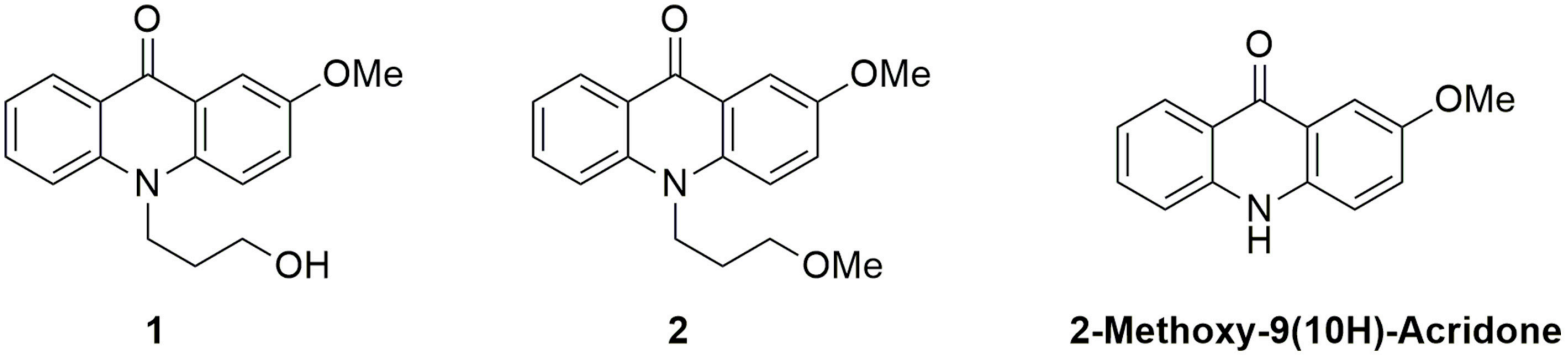
Structures of the N-substituted 2-methoxy-9-acridone derivatives employed in this work.

We focused our attention on the central moiety of 2-methoxy-9(10H)-acridone. To link this fluorescent moiety to other chemical groups and biomolecules, we added a 3-hydroxypropyl radical to the nitrogen atom of the chromophore. We obtained the N-(3-hydroxypropyl)-2-methoxy-9-acridone, **1**, in high yield as a suitable candidate for our studies (see Supplementary Materials for the synthesis reaction and characterization, and Supplementary Figures S1–S4). To investigate the effect of the hydroxy group of **1** on the proton transfer reactions, we prepared the N-(3-methoxypropyl)-2-methoxy-9-acridone, **2**, lacking the terminal hydroxy group, as described in the Supplementary Materials (and characterization in Supplementary Figures S5, S6). As a control, we also measured the precursor 2-methoxy-9(10H)-acridone (see Supplementary Materials). Figure 1 shows the chemical structures of the two acridone derivatives prepared in this study.

### Reagents

Reagents were purchased from Sigma-Aldrich at the maximum purity available (molecular biology quality). Solvents were spectroscopic grade. Aqueous solutions were prepared with MilliQ water and their pH were set with appropriate amount of acetic acid, sodium acetate, HNO_3_, and NaOH.

### Instrumentation

To absorption spectra of the 2-methoxy-9-acridone derivatives, we used a UV-Visible double-beam absorption spectrophotometer (Lambda 650; PerkinElmer, U.S.A.). Steady-state fluorescence spectra were collected on a FP-8300 spectrofluorometer (Jasco, Japan). All measurements were recorded using 10 × 10 mm cuvettes. The pH of each sample was measured immediately after recording each spectrum.

Fluorescence decay traces were recorded on a FluoTime 200 time-resolved fluorimeter (PicoQuant, Germany), with a TimeHarp 200 event tagging card working in single-photon timing mode. The excitation source was a 375-nm pulsed diode laser (LDH-375, PicoQuant) controlled by a PDL-800 driver (PicoQuant) and working at a repetition rate of 10 MHz. The fluorescence decay traces were collected at 440, 470, 500, and 530 nm, as the emission wavelengths, until 2 × 10^4^ counts were reached in the peak channel. For TRES acquisition, the fluorescence decay traces were obtained from 425 to 572 nm, every 3 nm. A constant period of time was employed to collect all the traces. For the cases when the laser power had to be changed for collecting a larger number of counts, the appropriate correction factors were applied to normalize the collection time.

### Data Analysis

The absorbance vs. pH curves were globally fitted to Equation (1), where *A*^λ^ is the absorbance at wavelength λ, *C*_*T*_ is the total concentration of the dye; *b* is the optical path; p*K*_*a*_ is the acidity constant, a globally adjustable parameter; and $\varepsilon_{HA}^{\lambda }$ and $\varepsilon_{A}^{\lambda }$ are the wavelength-dependent molar absorptivity coefficients of the protonated and deprotonated forms, respectively. Six different traces, obtained at the wavelengths of 405, 410, 415, 420, 425, and 450 nm, were employed in the fittings.

(1) $$\begin{array}{l} A^{\lambda }=C_{T}b\left(\varepsilon_{HA}^{\lambda }\frac{10^{-pH}}{10^{-pH}+10^{-pK_{a}}}+\varepsilon_{A}^{\lambda }\frac{10^{-pK_{a}}}{10^{-pH}+10^{-pK_{a}}}\right) \end{array}$$

The fluorescence intensity vs. pH curves were globally fitted to Equation (2), where *I*/*A* is the fluorescence intensity at a certain emission wavelength, λ_em_, after excitation at λ_ex_ and normalized by the absorbance at the excitation wavelength in each point; p$K_{a}^{*}$ is the globally shared excited-state acidity constant; and *f*_*HA*_ and *f*_*A*_ are proportionality factors directly related to the quantum yield of the protonated (HA) and deprotonated (A) forms, respectively.

(2) $$\begin{array}{l} \frac{I}{A}=f_{HA}\cdot \frac{10^{-pH}}{10^{-pH}+10^{-pK_{a}^{*}}}+f_{A}\cdot \frac{10^{-pK_{a}^{*}}}{10^{-pH}+10^{-pK_{a}^{*}}} \end{array}$$

All non-linear least squares fitting procedures were implemented in Origin Pro 9.0 (OriginLab Corp., U.S.A.).

The fluorescence decay traces were analyzed with the FluoFit software (PicoQuant) by using iterative deconvolution methods. The instrument response function (IRF) was obtained at the excitation wavelength from a scattering LUDOX solution. The fluorescence decay traces were fitted to a sum of two or three exponential decay components.

Time-resolved emission spectra (TRES) and species-associated emission spectra (SAEMS) were obtained though fluorescence decay traces collected over the 425–572 nm spectral range, with Δλ = 3 nm, and corrected for the same instrumental conditions and the same total acquisition time. TRES spectra were calculated using Equation (3) for total decay times of 0, 0.5, 0.8, 1, 1.2, 1.5, 3, 5, 8, 10, and 15 ns.

(3) $$\begin{array}{l} I_{\lambda }\left(t\right)=\;\sum_{i=1}^{n}p_{i}\cdot e^{\frac{-t}{\tau_{i}}} \end{array}$$

In Equation (3), *I*_λ_(*t*) is the time-dependent emission intensity at emission wavelength λ, fitted to an exponential model with *n* species; *p*_*i*_ is the amplitude of species *i* at λ; and τ_*i*_ is the corresponding decay time for species *i*.

The SAEMS represent the spectral contribution of each one of the species, *i*, estimated at each emission wavelength per Equation (4), in which the spectra need to be corrected by the corresponding total spectrum obtained at steady-state (*I*_*ss*__,λ_).

(4) $$\begin{array}{l} I_{i,\lambda }=\frac{p_{i}\cdot \tau_{i}}{\sum_{i=1}^{n}p_{i}\cdot \tau_{i}}I_{ss,\lambda } \end{array}$$

### Theoretical Calculations for the Absorption and Emission Spectra

To corroborate the empirical observations of compound **1**, theoretical simulations of the absorption and emission spectra of the protonated and deprotonated species of this molecule were performed. The calculation of every spectrum started with the optimization of the corresponding species by looking for the most stable geometry, followed by the determination of the vertical excitation energies and finally the emission wavelengths from the relaxation of the first excited state (S1) geometry and from the vertical emission to the ground state (GS). All the calculations were performed using the Gaussian 09 suite. The geometry optimizations of the different conformers and species of compound **1** were computed at the DFT CAM-B3LYP/6-31G^**^ level of theory. To verify that the found stationary points were true minima, the harmonic frequencies were computed. Time-dependent density functional theory was employed to describe the electronic transitions. Solvent effects were included by means of the integral equation formalism of the polarizable continuum method (IEF-PCM). All calculations were performed using the long-range corrected hybrid functional CAM-B3LYP and the 6-31G (d,p) basis set.

## Results

### Photophysical Properties and Acid-Base Equilibria

**Figure 2:**
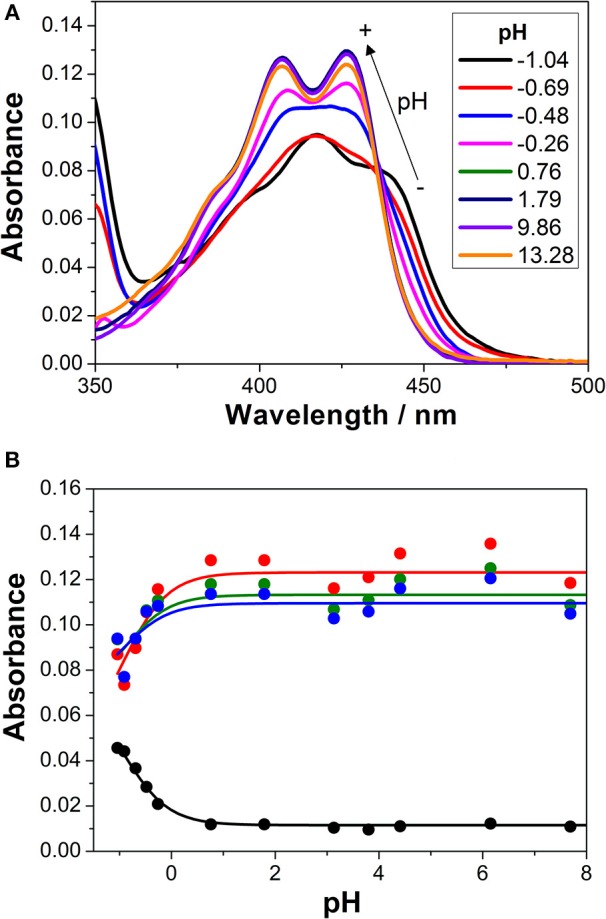
**(A)** Absorption spectra of **1** in aqueous solution at different pH-values. **(B)** Absorbance vs. pH curves at different wavelengths (blue = 415 nm, green = 420 nm, red = 425 nm, and black = 450 nm). The lines represent the results from the global non-linear curve fit to the general equilibrium and Beer's Law equations.

We first investigated the UV-visible absorption and fluorescence emission properties of **1** in aqueous solution, exploring its acid-base equilibria. In the pH range between 2 and 12, the absorption and emission spectra of the dye were practically invariable. Just when the pH was lowered below 0, spectral changes arose (Figure 2A). At a pH of approximately −1.0, the S0 → S1 absorption band displayed a maximum at 418 nm and several shoulders due to vibrational structures. Upon a pH increase, a broad band with two maxima at 407 and 426 nm appeared. The spectral changes at acidic pH-values are related to the protonation of the chromophore group of **1**. The hydroxyl radical in the 3-hydroxypropyl side group may also exhibit acid-base properties. However, since the group is not conjugated and far from the chromophoric moiety, its protonation-deprotonation equilibrium would not affect the spectral properties of the dye in the visible region. The equilibrium constant and p*K*_*a*_ of the detected protonation transition was obtained by globally fitting the absorbance vs. pH curves, collected at several wavelengths, to the general equations of Beer's Law and chemical equilibrium (Figure 2B). Six different *A* vs. pH traces were globally fitted, with the p*K*_*a*_ as a global adjustable parameter, using a non-linear least squares fitting procedure. We recovered a p*K*_*a*_ value of −0.94 ± 0.15, confirming the very weak basic character of acridone **1**, hence, the very strong acidic character of its protonated form.

**Figure 3:**
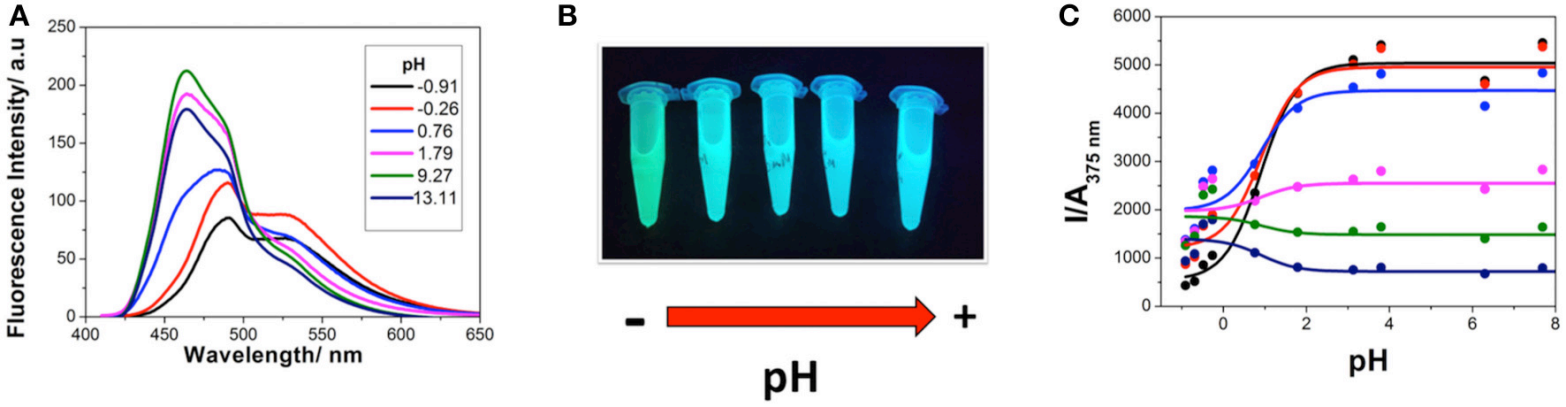
**(A)** Steady-state emission spectra (λ_ex_ = 400 nm) of **1** at different pH-values. **(B)** Solutions of **1** imaged under UV light irradiation at different pH-values, from 1.15 to 13.39. **(C)** Steady-state intensity normalized by absorbance vs. pH curves, at different emission wavelengths (black 460 nm, red 470 nm, blue 480 nm, magenta 500 nm, green 520 nm, and navy 550 nm), with λ_ex_ = 375 nm. Lines represent the results from the non-linear curve fit to Equation (2).

The investigation of the steady-state emission spectra of **1** at different pH-values also showed a similar behavior (Figure 3A). An almost invariable emission spectrum was found at pH-values higher than 2.0, with an emission maximum centered at 465 nm. As the pH of the environment was decreased, a new spectral shape arose, exhibiting an emission maximum at 490 nm and a notable secondary band centered at 530 nm. This shift to lower energies in the transitions upon protonation was also evident by the change in the color of the solution to a greener shade (Figure 3B). However, by inspecting the curves of intensity emission vs. pH, the protonation transition seemed to occur at higher pH-values than that obtained in the absorbance measurements. This result suggests that the basic form of species **1** at this acid-base equilibrium was stronger in the excited state, thus, leaving a weaker conjugate acid. This behavior opens up the possibility of excited-state dynamics; expressly, excited-state proton transfer (ESPT) reactions can take place when the acid-base properties of the molecule notably change upon excitation.

After being promoted to the excited state, fast protonation, or deprotonation reactions may occur during the lifetime of this excited state when a suitable proton donor-acceptor is present. In this case, hydronium ions and water molecules may act as the proton donor and acceptor, respectively. Provided the ESPT reaction is fast enough so that the apparent equilibrium is rapidly reached, the steady-state intensity emission is capable of describing the excited-state equilibrium constant, which is p*K*_a_^*^ (Alvarez-Pez et al., 2001). Therefore, the *I* vs. pH curves of **1**, collected at different emission wavelengths, were globally fitted, as described in section Data Analysis, and the p*K*_a_^*^ was estimated to be 0.91 ± 0.08 (Figure 3C). The increase in the p*K*_*a*_ value in the excited state confirms the enhanced basicity of the deprotonated form of **1**. This compound is one of the few examples of photobases, which have recently been suggested as potential candidates for light-driven pH-jump experiments (Sheng et al., 2018).

### Excited-State Dynamics: Coupled ESPT and Excimer Formation

**Figure 4:**
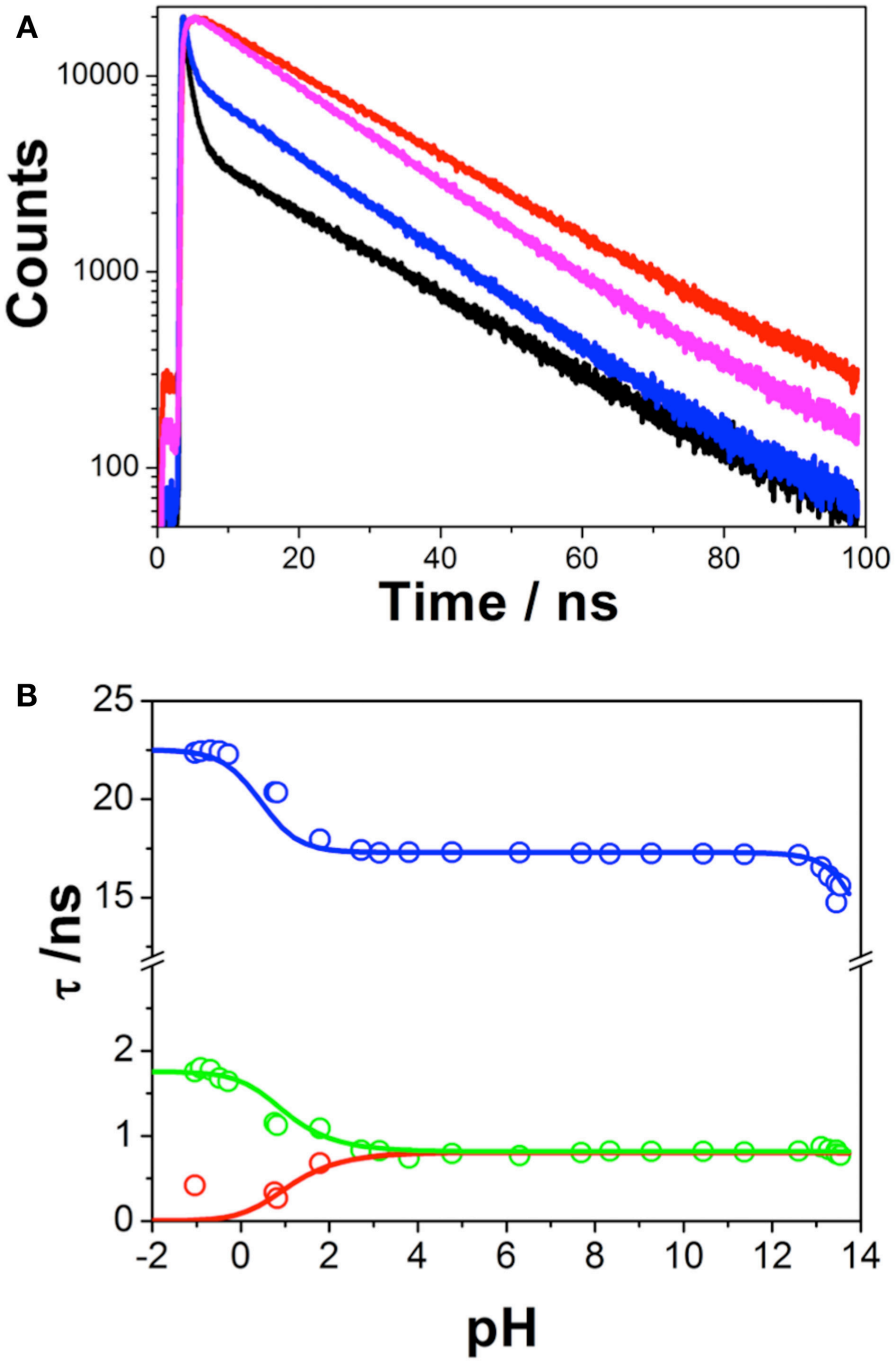
**(A)** Representative fluorescence decay traces of **1**, collected at pH 0.79 and λ_em_ = 440 nm (black) and λ_em_ = 530 nm (red), and pH 7.69 and λ_em_ = 440 nm (blue) and λ_em_ = 530 nm (magenta). **(B)** Fluorescence decay times from **1**, at different pH-values. Symbols represent experimental values from the global fits of the fluorescence decay traces, whereas lines represent the simulated decay times according to the kinetic scheme including an acid-base excited-state reaction and excimer formation.

**Figure 5:**
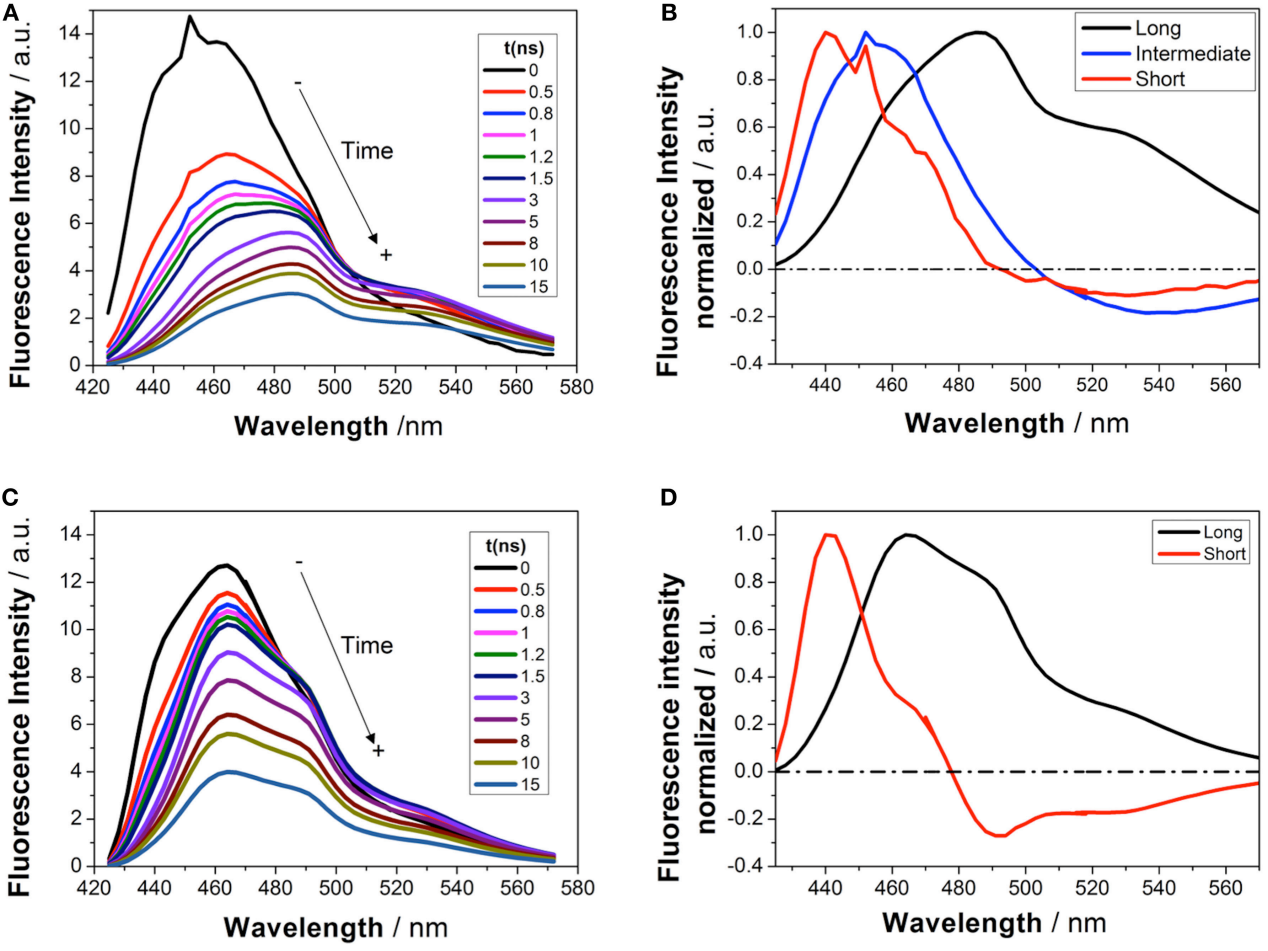
TRES **(A,C)** and SAEMS **(B,D)** of **1** in aqueous solution at pH 0.82 **(A,B)** and 13.45 **(C,D)**. The TRES spectra show the time evolution between 0 and 15 ns. The SAEMS show the emission spectrum associated with the longest (black), intermediate (blue), and shortest decay times (red).

Time-resolved fluorimetry represents the most suitable technique to investigate and characterize the excited-state dynamics of fluorescent probes. The fluorescence decay traces implicitly hold kinetic information about radiative and non-radiative deactivation processes, as well as reactions that alter the excited-state population of fluorescent molecules. Therefore, we employed this technique to explore the ESPT reaction of dye **1**. First, we focused on the pH range in which the ESPT reaction occurred, by collecting fluorescence decay traces at several λ_em_ to obtain more accurate estimations of the decay times from global fits. A simple ESPT reaction involving two prototropic species would show fluorescence decay traces with two different decay times as the solution of the corresponding excited-state kinetic equations (Boens and Ameloot, 2006). However, in the pH range between −1 and 2.5, we obtained fluorescence decay traces that required three different exponential terms (Figure 4). The shortest decay time, varying from 0.27 to 0.68 ns, was a rise time at long emission wavelengths, as it was characterized by a negative pre-exponential factor (Supplementary Figure S7). The presence of negative pre-exponentials is a unique feature of excited-state dynamics, indicating that the excited-state reaction results in a product with a higher emissive yield at the studied wavelength. The intermediate decay time decreased from 1.75 to 0.83 ns in the pH range between −1 and 2.5. Finally, the longest decay time varied from 22.36 to 17.41 ns, with a transition near the pH-value of approximately p*K*_a_^*^, as expected for an ESPT reaction. The TRES showed the transformation of the initially excited, emissive species into a species exhibiting a redshifted shoulder (Figure 5A). The analysis of the SAEMS when excited-state dynamics are present can provide information on which kinetic processes occur first, as well as the spectra of the deactivating and forming species. The three SAEMS of these fluorescence decay traces presented a complex dynamic situation (Figure 5B). The initially excited form, emitting in the blue edge (Figure 5B, red line), showed a transformation into a second form with a redshifted emission (the negative section of the fast decaying species). The species associated with the intermediate decay time (Figure 5B, blue line) still exhibited a negative region in the red-edge, indicating the formation of a different emissive species. This negative, redshifted shoulder correlated well with the emission shoulder found in the cation emission of **1** in very acidic media. Finally, the spectrum associated with the long decay time (Figure 5B, black line) represented the coupled decay of all the interrelated species. These results suggest two main points: (1) the coexistence of two different excited-state reactions, with different dynamics, and (2) the formation of the cationic species upon excitation, hence, a photobase behavior of the dye.

**Figure 6:**
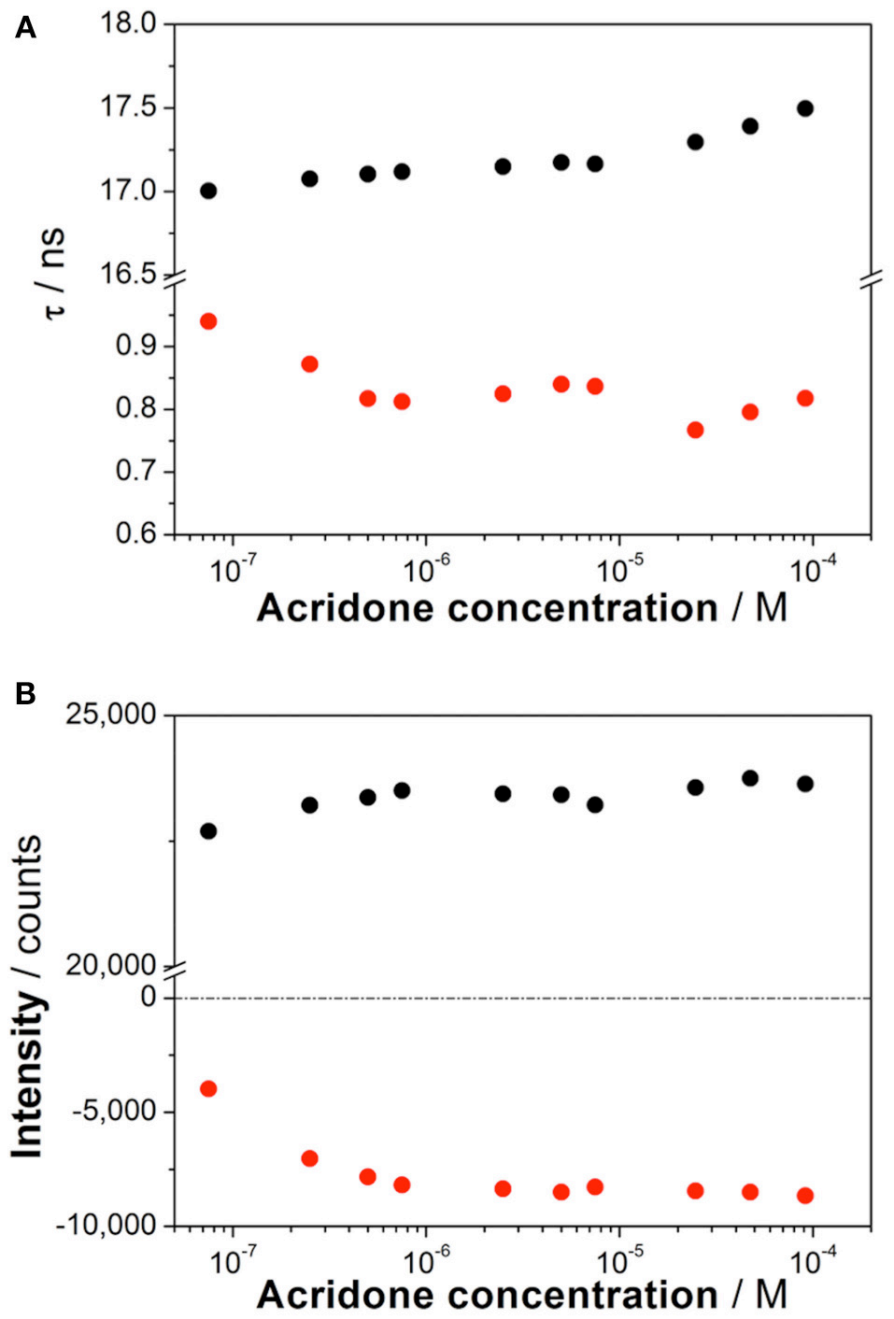
**(A)** Decay times from the fluorescence decay traces of increasing concentrations of **1** at pH 6.3. **(B)** Pre-exponential factors of the long (black) and short (red) decay times from the decay traces of increasing concentrations of **1** at pH 6.3 (λ_em_ = 530 nm).

To investigate the coexistence of the two excited-state reactions, we moved to near-neutral pH-values. When the pH is far from the range at which the ESPT reaction is feasible, one would expect mono-exponential decay traces from a single emitting species. In contrast, for the pH range from 2.5 to 13.5, we found a biexponential decay behavior. Importantly, the shortest decay time was a rise time in the emission wavelengths beyond 500 nm, where a secondary emission band was detected in the steady-state emission spectra. This result indicates that the secondary band formed upon excitation, with fast kinetics. This was also confirmed by studying the TRES and SAEMS under the same conditions (Figures 5C,D, and Supplementary Figure S8). The presence of an isoemissive point in the TRES (Figure 5C) clearly indicated the transformation of the initially excited species into a redshifted emitting form (the negative region in the SAMES associated with the fast decay time, Figure 5D). The appearance of a redshifted, broad band is a typical feature of an excimer-like arrangement, whereby dimers are formed during the excited state (Lakowicz, 2006). Hence, our hypothesis is that the excited-state dynamics found for compound **1** are due to excimer formation. To test this hypothesis, we collected fluorescence decay traces at different total concentrations of **1**, as the dynamics of excimer formation must be concentration-dependent. Indeed, the short rise time decreased from 0.94 ns at 7.5 × 10^−8^ M to 0.77 ns at 2.5 × 10^−5^ M (Figure 6A), indicating that the excimer formation became faster with the concentration increments. Likewise, the negative pre-exponential also gained statistical weight with increasing concentrations of **1** (Figure 6B), confirming that the excimer formation reaction occurred to a greater extent. The excimer formation hypothesis was further supported by theoretical calculations, in which the most likely geometry of the excimer was obtained (see section Theoretical Calculations). The appearance of a rise time in the fluorescence decay traces is an unequivocal feature that the emitting species at the redshifted band is in fact formed mainly in the excited-state, and hence it is an excimer. Nevertheless, the π-rich structure of the acridone moiety may promote aggregation by π-stacking also in the ground state. In order to test this, we focused on the ground-state, absorption spectrum of **1**, at different concentrations. The absorption spectra remained invariable in shape, and the absorbance strictly followed the Beer's Law (see Supplementary Figure S9). The concentration-independence of the absorption spectrum does not support the formation of dimers or aggregates in the ground state. Hence, we can conclude that the interaction occurs mainly involving an excited monomer.

Finally, at high pH-values, a decrease in the long decay time was detected (Figure 4B). This decrease was accompanied by a decrease in the total emission intensity. These features could be interpreted as an additional acid-base reaction occurring under highly basic conditions (Schulman and Sturgeon, 1977); however, the absence of acidic hydrogen atoms or electron acceptor positions make this hypothesis unlikely. Likewise, the absorption spectrum of **1** did not change at these pH-values. Therefore, we concluded that this effect was mainly caused by an OH^−^-mediated quenching of the excited state.

Hence, this situation represents a challenging kinetic system, with coupled excited-state dynamics, involving both an ESPT reaction and an excimer formation reaction, together with an additional quenching reaction at high pH. Since the excited-state dynamics were more complex than expected, further investigations were required to gain insight into the system. Our approach to fully analyze and solve the excited-state dynamics of this system consisted of a GCA methodology, which included all the emissive species found (see section Compartmental Analysis of the Coupled Excited-State Dynamics).

To rule out the possibility of the hydroxy group in the N-propyl chain being involved in an intramolecular proton transfer, we synthesized N-(3-methoxypropyl)-2-methoxy-9-acridone, **2**, as a control. In **2**, the terminal hydroxyl radical has been converted into a methoxy radical, so that proton transfer reactions are hindered at this position. The spectroscopic features (absorbance and steady-state emission spectra) and the acid-base behavior of **2** were very similar to those of **1** (see Supplementary Figure S10). The obtained ground state p*K*_*a*_ was −0.80 ± 0.13 for the cation → neutral transition, whereas the recovered excited-state p$K_{a}^{*}$ value was 1.04 ± 0.04. Likewise, compound **2** also displayed a biexponential behavior in the fluorescence decay traces at pH-values between 2.5 and 13.5, with a short rise time of 0.8 ns (Supplementary Figure S10C), supporting the efficient formation of an excited-state excimer. The emission of **2** also exhibited a quenching caused by hydroxyl groups at high pH-values. All these results illustrate the similarities between both dyes. Indeed, this was expected, as the hydroxyl in **1** and the methoxy in **2** and the chromophore moiety are not conjugated while being sufficiently separated to not cause a measurable effect on the dynamics of the first excited state.

### Theoretical Calculations

**Figure 7:**
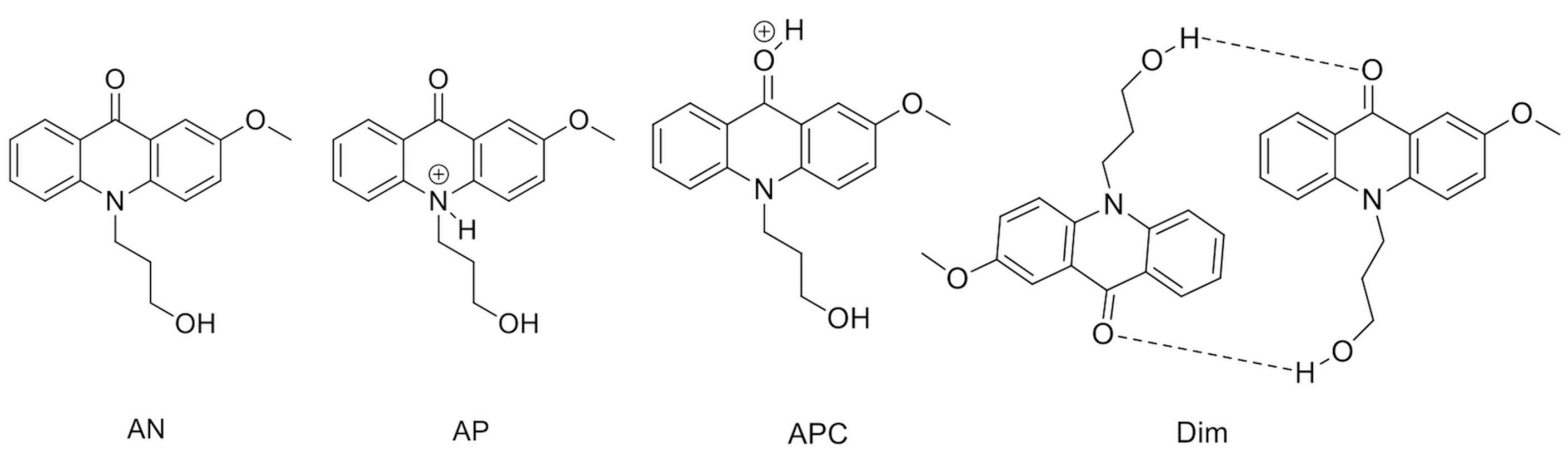
Different structures of **1** studied by theoretical calculations.

**Figure 8:**
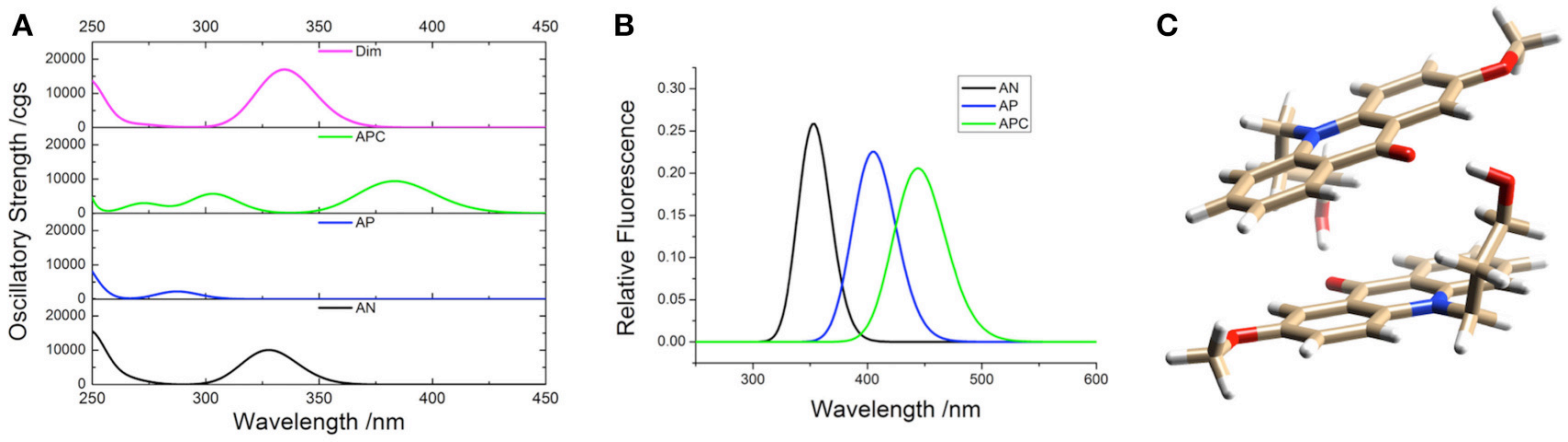
Theoretical absorption **(A)** and emission **(B)** spectra from the gas phase of the different forms of **1**: neutral (AN, black), protonated at the nitrogen (AP, blue), protonated at the carbonyl-oxygen (APC, green), and dimer (Dim, magenta). **(C)** Optimized geometry of the dimer (Dim) in the gas phase, with the following color codes: white, H atoms; light brown, C atoms; red, O atoms; blue, N atoms.

To support the conclusions drawn about the photophysical properties of **1**, we performed theoretical calculations, minimizing the energy and estimating the most likely absorption and emission transitions in the gas phase of the forms depicted in Figure 7 (see Tables S1, S2). The first point to be investigated was elucidating the actual protonation position. We considered two possibilities for protonation: at the acridone nitrogen (AP) or at the carbonyl oxygen (APC). The theoretical calculations demonstrated that a larger negative charge was concentrated at the carbonyl oxygen, making this position more likely to be protonated. Likewise, when comparing the calculated absorption and emission spectra (Figure 8), the APC form was the one that effectively showed a redshifted HOMO → LUMO transition, as we found experimentally at low pH-values. Hence, we concluded that protonation is most likely at the carbonyl group.

We also explored the potential structure of a dimer (Dim in Figure 7) to simulate the excimer formation. After optimizing the geometry (Figure 8C), the calculated absorption spectrum of Dim exhibited a redshift compared to that of the neutral AN, but not to energies as low as that of the cationic form (Figure 8A). This result is in agreement with the experimental data, especially the TRES and SAEMS spectra (Figure 5). Nonetheless, we could not obtain a simulated emission transition for Dim in the gas phase. However, we obtained the emission transition for Dim in water, which exhibited a redshift compared to that of the AN and was in agreement with the experimental results. Further work to include general and specific solvent effects on these simulations is in progress.

### Compartmental Analysis of the Coupled Excited-State Dynamics

The qualitative analysis of the spectroscopic and kinetic results, supported by the theoretical calculations, suggested the presence of coupled ESPT and excimer formation reactions, and a quenching process mediated by OH^−^ ions. To solve such challenging dynamics, we employed the GCA approach. This approach shows superior performance in the analysis of the complex excited-state dynamics of fluorescent dyes, in terms of reliability and identifiability of the obtained results (Boens and Ameloot, 2006).

**Figure 9:**
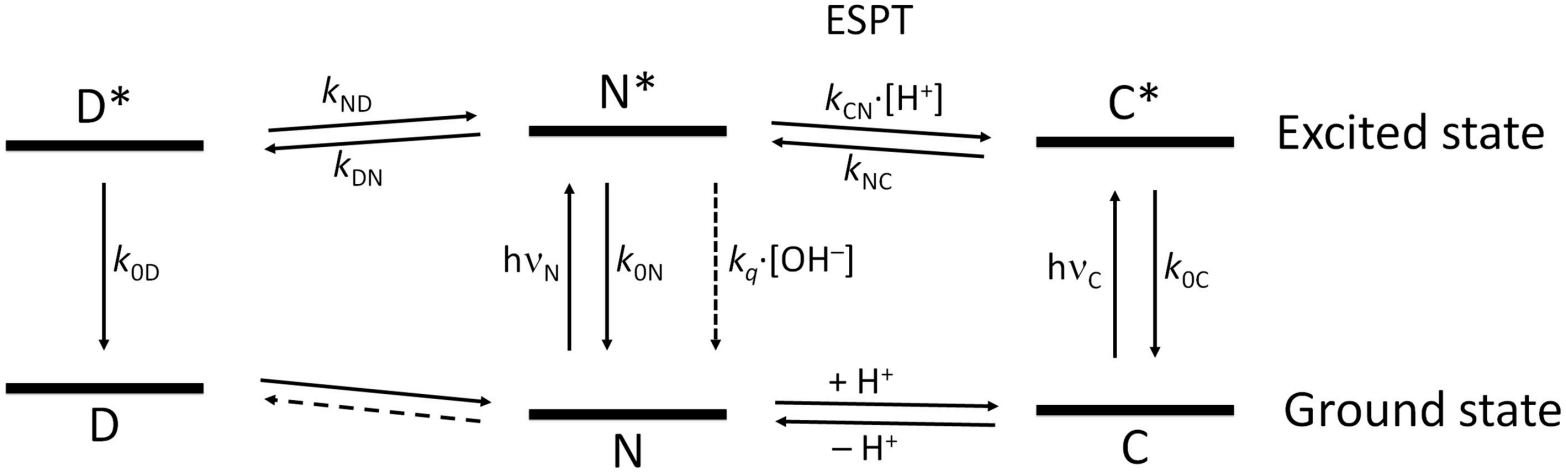
Scheme of the tricompartmental excited-state system of the N-substituted 2-methoxy-9-acridones employed in this work. The different species present are: D, the excimer; N, the neutral prototropic form; and C, the acid form. The kinetic scheme considers the formation and dissociation of the excimer (*k*_DN_ and *k*_ND_, respectively), the ESPT reaction between N and C (*k*_CN_ for the protonation, and *k*_NC_ for the deprotonation), the radiative deactivation processes (*k*_0D_, *k*_0N_, and *k*_0C_), and the nonradiative quenching of N mediated by OH^−^ ions (*k*_*q*_). We did not find experimental evidence of the formation of dimers in the ground state, therefore, this reaction is represented by a dashed line. The vertical distance between the ground and the excited states for each species is proportional to the experimental bandgap based on the emission spectra.

Figure 9 shows the overall kinetic scheme that we considered herein to analyze the excited-state dynamics of the N-modified 2-methoxy-9 acridones, specifically compound **1**. The kinetic scheme involves the excited-state protonation (*k*_CN_) and deprotonation (*k*_NC_) of the neutral (N) and acidic (C) forms, respectively; as well as the formation (*k*_DN_) and dissociation (*k*_ND_) of a neutral excimer (D). The excimer formation rate, *k*_DN_, would be concentration-dependent; however, as a single concentration was studied globally, it can be considered a pseudofirst-order constant.

When the system in solution is excited by an infinitesimal δ-pulse of light at time *t* = 0, the time evolution of the different excited species gathered in the vector **x**(*t*), in which each element represents the concentration of one of the excited species (D, N, or C), follows the differential equation

(5) $$\begin{array}{l} \dot{\text{x}}\left(t\right)=\text{A}\cdot \text{x }\left(t\right) \end{array}$$

where $\dot{\text{x}}\left(t\right)$ is the time derivative of vector **x**(*t*) and **A** is the 3 × 3 compartmental matrix, which contains all the kinetic processes of each species (Orte et al., 2005b; Boens and De Schryver, 2006). In this particular case, the matrix **A** has the form

(6) $$\begin{array}{l} \text{A}=\left(\begin{array}{ccc} -\left(k_{0D}+k_{ND}\right) & k_{DN} & 0\\ k_{ND} & -\left(k_{0N}+k_{DN}+k_{CN}\cdot \left[H^{+}\right]+k_{q}\left[OH^{-}\right]\right) & k_{NC}\\ 0 & k_{CN}\cdot \left[H^{+}\right] & -\left(k_{NC}+k_{0C}\right) \end{array}\right) \end{array}$$

In a coupled, compartmental system such as this, the fluorescence decay traces collected at different emission wavelengths would be described by three different decay times τ_*i*_ (unless non-emissive species are present). The τ_*i*_ values are related to the eigenvalues of matrix **A**, γ_*i*_, through Equation (7).

(7) $$\begin{array}{l} \gamma_{i}=-\frac{1}{\tau_{i}} \end{array}$$

If an analytical expression for the eigenvalues γ_*i*_ could be obtained, the dynamic system could be resolved by a non-linear least squares fitting to the experimental data. However, the intricacy of the compartmental matrix prevents obtaining an explicit expression for the eigenvalues. Hence, we designed an iterative process to assess the value of each kinetic rate constant in the model.

The first step involved analyzing the limiting values of the eigenvalues at very low pH. In this limit, the long decay time is given by ${\left(k_{0C}\right)}^{-1}$, and the intermediate decay time is given by ${\left(k_{0D}+k_{ND}\right)}^{-1}$. Therefore, by using the experimental values of the decay times at very low pH, we obtained *k*_0C_ = 0.044 ns^−1^ and *S*_1_ = *k*_0D_ + *k*_ND_ = 0.570 ns^−1^. Then, we focused on the pH region between 3 and 11, in which only two invariable decay times were experimentally obtained (Figure 4B). In this region, the pH is sufficiently high so that the acidic form does not intervene, and low enough so that the hydroxyl-mediated quenching is negligible. Under these experimental conditions, the compartmental matrix can be reduced as follows.

(8) $$\begin{array}{l} \text{A}=\left(\begin{array}{cc} -\left(k_{0D}+k_{ND}\right) & k_{DN}\\ k_{ND} & -\left(k_{0N}+k_{DN}\right) \end{array}\right) \end{array}$$

This bicompartmental model indeed predicts fluorescence decay traces with two pH-independent decay times, as we experimentally observed. However, as there are four different rate constants and only two eigenvalues, the system cannot be unequivocally determined. We then set an initial guess for *k*_0D_ (so that *k*_ND_ was immediately defined through *S*_1_) and employed the experimental decay times to determine *k*_0N_ and *k*_DN_, as there were only two remaining unknowns. With these guesses for the rate constants in the near-neutral pH region, we moved to the acidic region and employed the experimental values of the three decay times to estimate *k*_NC_ and *k*_CN_, taking into account the relation between the sum of the eigenvalues and the rate constants (Equation 9) (Boens and De Schryver, 2006) and the experimental value of p*K*_a_^*^ (Equation 10).

(9) $$\begin{array}{l} \underset{i}{\sum }\gamma_{i}=-S_{1}-k_{0N}-k_{DN}-k_{0C}-k_{NC}-k_{CN}\cdot \left[H^{+}\right] \end{array}$$

(10) $$\begin{array}{l} K_{a}^{*}=\frac{k_{NC}}{k_{CN}} \end{array}$$

With the new values for *k*_0N_, *k*_DN_, *k*_NC_, and *k*_CN_, we obtained a new guess for *k*_0D_ (and hence, the linearly dependent *k*_ND_) by performing a non-linear curve fitting of the experimental values of the long decay time. Then, we repeated the process iteratively until consistent solutions for all the rate constants were achieved, as depicted in Supplementary Figure S11.

Finally, we moved to the high pH region to assess the value for the quenching constant *k*_*q*_ over the neutral form. For this pH region, the bicompartmental matrix has the form

(11) $$\begin{array}{l} \text{A}=\left(\begin{array}{cc} -\left(k_{0D}+k_{ND}\right) & k_{DN}\\ k_{ND} & -\left(k_{0N}+k_{DN}+k_{q}\left[OH^{-}\right]\right) \end{array}\right) \end{array}$$

Analytical expressions for the two eigenvalues can be obtained, with each one assuming the positive or negative root of the following equation (where *S*_1_ = *k*_0D_ + *k*_ND_ and *S*_2_ = *k*_0N_ + *k*_DN_).

(12) $$\begin{array}{l} \gamma_{1,2}=\frac{-S_{1}-S_{2}-k_{q}\left[OH^{-}\right]\pm \sqrt{k_{q}\left[OH^{-}\right]\left[k_{q}\left[OH^{-}\right]-2\left(S_{1}+S_{2}\right)\right]+{\left(S_{1}+S_{2}\right)}^{2}-4\left({k_{0N}S}_{1}+{k_{0D}S}_{2}-k_{0D}k_{0N}\right)}}{2} \end{array}$$

We globally fitted the two experimental decay times, obtained between pH 6.3 and 13.5, to Equation (12), leaving as fixed parameters the already known rate constants, and as a globally adjustable parameter, the value of *k*_*q*_. We obtained a value of 0.035 M^−1^ ns^−1^ for this constant.

Table 1 gathers the recovered values of all the rate constants for the excited-state reaction depicted in Figure 9. With all these rate constants well-defined, we could predict and simulate all three decay times through the eigenvalues of the complete tricompartmental matrix **A** (Equation 6). These simulations can be seen as the lines in Figure 4B, in which one can observe the perfect agreement between the predicted and the experimental values.

**Table 1:** Equilibrium constants and kinetic rate constants of acridone **1** in aqueous solution, according to the excited-state dynamics depicted in Scheme 3.

**Constant**	**Value**
*k*_0D_ /ns^−1^^(a)^	0.015 ± 0.070
*k*_0N_ /ns^−1^	0.113 ± 0.004
*k*_0C_ /ns^−1^	0.044 ± 0.002
*k*_DN_ /ns^−1^^(b)^	0.624 ± 0.021
*k*_ND_ /ns^−1^	0.555 ± 0.025
*k*_NC_ /ns^−1^	1.178 ± 0.023
*k*_CN_ /M^−1^ ns^−1^	9.571 ± 1.870
*k_*q*_* /M^−1^ ns^−1^	0.035 ± 0.003
p*K_*a*_*	−0.94 ± 0.15
pK_a_*	0.91 ± 0.08

(a) The associated error for k_0D_ is very large, so that it cannot be well defined. However, what it is well defined is the sum S_1_ = k_0D_ + k_ND_ = 0.57 ± 0.03.

(b) *The k_DN_ rate constant is a pseudofirst-order rate for the excimer formation at a constant concentration*.

## Discussion

Modified acridone derivatives are among the most widely used compounds with biological activity, due to their small size and easy incorporation into cellular compartments and to their interaction with nucleic acids. With these interesting applications, an in-depth knowledge of their underlying photophysical behavior is mandatory for their usage as antitumoral agents (Cholewinski et al., 2011), in photodynamic therapy or as laser and OLED active media (Sharma et al., 2016; Pander et al., 2018). Herein, we analyzed in-depth the acid-base and spectroscopic properties, as well as the excited-state dynamics, of two N-substituted 2-methoxy-9-acridone derivatives.

The acid-base ground-state equilibria of compounds **1** and **2** are in agreement with previous data on 2-methoxy-9(10H)-acridone (Schulman and Sturgeon, 1977), i.e., a negative ground-state p*K*_*a*_ and a notable decrease in the acidity constant upon excitation. In our case, an increase of 1.85 units of p*K*_*a*_ was found upon excitation of **1**, and 1.84 for compound **2**. These effects are clear examples of photobase behavior, a significantly less studied effect compared to that of photoacids (Sheng et al., 2018). The protonation position was established to occur at the O-atom of the carbonyl group, which acts as an electron acceptor group, whereas the N-atom has an electron donating behavior. This feature as well as the potential steric hindrance of the substituted N-atom make the O-atom the most likely position for protonation (Nikolov et al., 1998). We found additional support for this conjecture from the theoretical calculations (Figure 8), which evidenced that protonation at the N-atom would result in a blueshifted absorption with respect to that of the neutral. In contrast, the simulated results for protonation at the carbonyl were in agreement with the experimental the redshifted absorption and emission spectra of the cationic species.

Interestingly, when compounds **1** and **2** were dissolved at near-neutral pH, far from acidic media in which the cation may be involved, striking excited-state dynamics were found (Figures 4–6). We assigned this result to the formation of an emissive excimer. In a previous report, intermolecular hydrogen bonding was found as the mechanism behind the stabilization of dimers in 4-acridinecarboxamide imines, as suggested by AM1 calculations (Fröhlichová et al., 2009). However, this action was driven by the proton at the acridone N-atom, and in our case, substitution at this position prevented such hydrogen bonding. We also ruled out the possibility of an intramolecular proton transfer involving the hydroxyl radical at the N-hydroxypropyl substituent in **1** with two arguments: (1) theoretical calculations for intramolecular hydrogen bonding could not be optimized, and (2) the results from **2**, lacking the terminal OH group, were practically identical to those from **1**. We also explored the dependency of the formation of the excimer on concentration, and found that the short-lived decay rate became faster and gained statistical weight as the concentration was increased (Figure 6), supporting the idea of an intermolecular process. Likewise, we ruled out the possibility of ground-state dimerization or aggregation (Supplementary Figure S9). Therefore, we confirmed an excited-state dimerization, which must be driven by π-stacking interactions. Furthermore, we demonstrated, with data from compound **2**, that such behavior is common and may be general for the N-substituted-9-acridone moiety.

At high pH-values, we found a decrease in the long fluorescence decay time (Figure 4). This result could be interpreted in terms of an additional p*K*_*a*_, following further deprotonation, as happens in 9-(10H)-acridone and 2-methoxy-9(10H)-acridone (Schulman and Sturgeon, 1977). To test this conjecture, we also obtained the absorption and emission spectra of 2-methoxy-9(10H)-acridone, in which the N position of the chromophoric moiety is not substituted. An additional deprotonation event was evident since both the absorption and emission spectra exhibited clear changes, with the appearance of a blueshifted shoulder, and a clearer vibronic structure at high hydroxyl concentrations (Supplementary Figure S12). This confirmed the potential deprotonation of the N-position, and the formation of an anionic species, as previously postulated (Schulman and Sturgeon, 1977). However, this position is hindered in compounds **1** and **2**. Therefore, the decreases in fluorescence intensity and fluorescence lifetime cannot be caused by deprotonation and must be caused by a hydroxyl-mediated quenching, such as that proposed to affect lanthanide luminescence emissions (Yan et al., 1995; Orlovskii et al., 2016).

With all these data, we established intricate excited-state dynamics (Figure 9) in which coupled ESPT reactions and excimer formation-dissociation coexisted with a hydroxyl-mediated quenching process. This coupled kinetic system was approached with a tricompartmental approach and a thorough iterative fitting protocol. The application of GCA to the study of the complex excited-state dynamics of fluorescent dyes yielded a remarkable improvement in terms of the capabilities and reliability of the obtained results. From simple two-state excited-state reactions (Boens and Ameloot, 2006) to buffer-mediated ESPT reactions (Boens et al., 2004; Crovetto et al., 2004; Orte et al., 2005a) and complex three-state systems (Orte et al., 2005b), the GCA provided invaluable tools to gain in-depth information on those photophysical systems. Such studies have also helped in the experimental design of experiments suitable enough to obtain unique values for all the rate constants involved in the dynamic systems (Boens et al., 2004; Boens and Ameloot, 2006). To fully solve the system, at very high acidic concentrations, the reduction of available water molecules had to be considered, as previously done in the study of the superphotoacid 2′,7′-difluorofluorescein (Orte et al., 2005b). The complex excited-state dynamics found at acidic pH were successfully modeled, and all the rate constants were defined. The excited-state deprotonation rate of the cationic form was lower than those of the superphotoacids (Tolbert and Solntsev, 2002), indicating that the contribution of the cationic form was weaker in the excited-state. Therefore, the 2-methoxy radical induces a greater electron density to the acridone moiety, which usually results in a stronger base character than that of the unsubstituted acridones (Schulman and Sturgeon, 1977). This notion is supported by the quantum mechanics calculations. In the excited state, the cationic form exhibits an increased electron density on the central oxygen of the acridone moiety, as evidenced in the molecular orbital representations. This position is where the cation is protonated, hence, this shift of the electron density toward the protonable group in the excited-state makes it a weaker acid. For the rest of the pH range, the kinetic rate constants found (Table 1) fully describe the excited-state dynamics of the system. Interestingly, we tried other kinetic schemes different to Figure 9 and found that none were suited to fully explain the system. For instance, we tried a simpler scheme in which the dissociation of the excimer was not considered (*k*_ND_ → 0). In such a case, the model predicted a pH-independent intermediate decay time at low pH-values. This was not in agreement with our observations; therefore, the presence of a dissociation pathway of the excimer was justified, and the validity of Figure 9 over the entire pH range was confirmed.

In conclusion, N-substituted 2-methoxy-9-acridone derivatives exhibit a rich, dynamic photophysical behavior. Hence, not only are they interesting as potential long-lifetime sensors for FLIM applications, but their strong photobase behavior opens up possibilities for their use in light-driven pH-jump experiments.

## Data Availability

Datasets are available on request. The raw data supporting the conclusions of this manuscript will be made available by the authors, without undue reservation, to any qualified researcher.

## Author Contributions

PH-F, SR, and DM: syntheses and characterization of new compounds. SC: theoretical calculations. DM and JC: design and supervision of the syntheses of new compounds. MG-G and EG-F: spectroscopy experiments. MG-G and AO: Excited-state dynamics and GCA analyses. JA-P, MG-G, and AO: writing of the original draft with input from all authors, who read and approved the submitted version.

### Conflict of Interest Statement

The authors declare that the research was conducted in the absence of any commercial or financial relationships that could be construed as a potential conflict of interest.
