# Supplementary material for: Coupled Excited-State Dynamics in N-Substituted 2-Methoxy-9-Acridones
Source: Front Chem. 2019 Mar 12;7:129. doi: 10.3389/fchem.2019.00129 (PMC6422897; doi:10.3389/fchem.2019.00129)
Supplement: Supplementary file 1 [file Data_Sheet_1.pdf]

Supplementary Material

Coupled Excited-State Dynamics in N-substituted 2-Methoxy-9-Acridones

M. Carmen Gonzalez-Garcia[1], Pilar Herrero-Foncubierta[1,2], Silvia Castro[2], Sandra Resa[2], Jose M. Alvarez-Pez[1], Delia Miguel[1], Juan M. Cuerva[2], Emilio Garcia-Fernandez[1], Angel Orte[1,*]

[1] Departamento de Fisicoquimica. Facultad de Farmacia. Unidad de Excelencia en Quimica Aplicada a Biomedicina y Medioambiente (UEQ). Universidad de Granada. Campus Cartuja, 18071 Granada (Spain)

[2] Departamento de Quimica Organica. Facultad de Ciencias. Unidad de Excelencia en Quimica Aplicada a Biomedicina y Medioambiente (UEQ). Universidad de Granada. Campus Fuentenueva, 18071 Granada (Spain)

* **Correspondence:** Angel Orte: angelort@ugr.es

Table of Contents

1 Synthesis and characterization of the compounds 3
1.1 General details 3
1.2 Synthesis of precursor reagents 3
1.2.1 Synthesis of compound II 3
1.3 Synthesis of compounds 1 and 2 4
1.4 ^{1}H-NMR and ^{13}C-NMR spectra of new compounds 5
Supplementary Figure S1. ^{1}H NMR (500 MHz, $CDCl_3$) spectrum of compound II. 5
Supplementary Figure S2. ^{13}C NMR (126 MHz, $CDCl_3$) spectrum of compound II. 6
Supplementary Figure S3. ^{1}H NMR (600 MHz, MeOD) spectrum of compound 1. 6
Supplementary Figure S4. ^{13}C NMR (151 MHz, MeOD) spectrum of compound 1. 7
Supplementary Figure S5. ^{1}H NMR (500 MHz, MeOD) spectrum of compound 2. 7
Supplementary Figure S6. ^{13}C NMR (126 MHz, MeOD) spectrum of compound 2. 8
2 Supplementary Figures and Tables 9
2.1 Supplementary Figures 9

Supplementary Figure S7. Pre-exponential factors of the long (A) and the short (B) decay times of 1 as a function of pH at the emission wavelengths 440 (black), 470 (red), 500 (blue), and 530 nm (magenta). 9

Supplementary Figure S8. TRES (A) and SAEMS (B) of 1 in aqueous solution at pH 6.15. The TRES spectra show the time evolution between 0 and 15 ns. The SAEMS show the emission spectrum associated to the longest (black) and the shortest decay time (red). 9

Supplementary Figure S9. Absolute (A) and normalized (B) absorption spectra of different concentrations of 1 in aqueous solution at pH 6.30. (C) Plot of the absorbance at 400 nm, from the spectra of panel (A), versus the concentration of 1, showing an excellent linear relation, according to Beer's Law. 10

Supplementary Figure S10. (A) Absorption spectra, (B) steady-state emission spectra (λ_{ex} = 400 nm), and (C) fluorescence decay times of 2 in aqueous solution at different pH values. 10

Supplementary Figure S11. Iterative fitting process to recover all the kinetic rate constants for the dynamic excited-state behavior of 1 in aqueous solution. 11

Supplementary Figure S12. (A) Absorption spectra and (B) steady-state emission spectra (λ_{ex} = 400 nm) of 2-methoxy-9(10H)-acridone in aqueous solution and different pH values. (C) Normalized emission spectra of 1 (black), 2 (red), and 2-methoxy-9(10H)-acridone (blue) at pH > 13. 11

2.2 Supplementary Tables 12

2.2.1 Table S1. DFT energies and number of imaginary frequencies of the studied forms in the gas phase. 12

2.2.2 DFT Geometries in the gas phase 13

2.2.2.1 Table S2. Representative bond distances and angles from the DFT-optimized geometries of the different studied species in the gas phase. 13

2.2.2.2 AN – atom coordinates 15

2.2.2.3 AP – atom coordinates 16

2.2.2.4 APC – atom coordinates 17

2.2.2.5 Dim – atom coordinates 18

3 References 22

1 Synthesis and characterization of the compounds

1.1 General details

All reagents and solvents (CH_2Cl_2, ethyl acetate (EtOAc), hexane, CH_3CN and methanol (MeOH)) were purchased from standard chemical suppliers and used without further purification. To ensure dryness of tetrahydrofuran (THF), it was freshly distilled over Na/benzophenone. Anhydrous solvents (dimethylformamide (DMF), CH_2Cl_2 and diethyl ether (Et_2O)) were purchased from standard suppliers. Thin-layer chromatography (TLC) analysis was performed on aluminium-backed plates coated with silica gel 60 (230-240 mesh) with F_{254} indicator. The spots were visualized with UV light (254 nm and 360 nm) and/or stained with phosphomolybdic acid (10% ethanol solution) and subsequent heating. All chromatography purifications were performed with silica gel 60 (230-400 mesh). NMR spectra were measured at room temperature. ^1H NMR spectra were recorded at 500 or 600 MHz. Chemical shifts are reported in ppm using residual solvent peak as reference ($CHCl_3$: δ = 7.26 ppm, CH_3OH: δ = 3.31 ppm). Data are reported as follows: chemical shift, multiplicity (s: singlet, d: doublet, t: triplet, q: quartet, quint: quintuplet, m: multiplet, dd: doublet of doublets, dt: doublet of triplets, td: triplet of doublets, bs: broad singlet), coupling constant (*J* in Hz) and integration; ^{13}C NMR spectra were recorded at 126 or 151 MHz using broadband proton decoupling and chemical shifts are reported in ppm using residual solvent peaks as reference ($CHCl_3$: δ = 77.16 ppm, CH_3OH: δ = 49.00 ppm). Carbon multiplicities were assigned by distortionless enhancement by polarization transfer (DEPT) techniques. High-resolution mass spectra (HRMS) were recorded using EI at 70eV on a Micromass AutoSpec (Waters) or by ESI mass spectrometry carried out on a Waters VG AutoSpec mass spectrometer. The following known compounds were prepared as previously described, isolated as pure samples and showed NMR spectra identical to reported data: **I** (Chen and Hong, 2012), **III** (Pintér and Klussmann, 2012).

1.2 Synthesis of precursor reagents

1.2.1 Synthesis of compound II

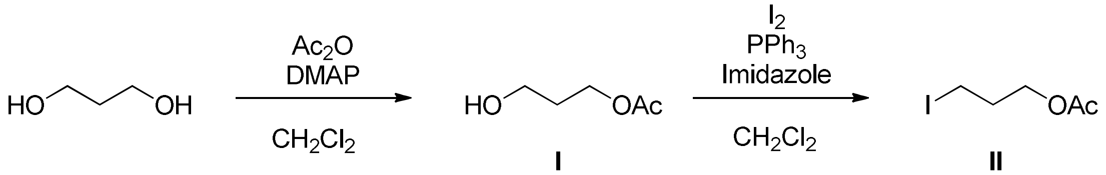

<u>Compound I</u>. To a solution of 1,3-propanediol (6.00 g, 0.08 mol) in CH_2Cl_2 (30 mL), DMAP (10.51 g, 0.09 mol) was added and subsequently acetic anhydride (8 mL, 0.09 mol) was added dropwise. The mixture was stirred for 2 h at room temperature. Silica gel was then added and the solvent was removed. The crude was purified by flash chromatography (SiO_2, Hexane/EtOAc 6:4) to give compound **I** (4.01 g, 43%) as a colorless oil. ^1H and ^{13}C NMR spectra matched to those of the reported ones (Chen and Hong, 2012).

<u>Compound II.</u> To a solution of I_2 (12.92 g, 0.05 mol) in anhydrous CH_2Cl_2 (50 mL), triphenylphosphine (12.92 g, 0.05 mol) was added thus giving a brown-yellow solution. Then, imidazole (7.63 g, 0.11 mol) was added, changing the colour to light yellow. Next, compound **I** (4.01 g, 0.03 mol) was added and the mixture was stirred at room temperature until consumption of the

starting material (checked by TLC, around 1-2 h). Silica gel was then added and the solvent was removed. The crude was purified by flash chromatography (SiO_2, Hexane/EtOAc 9:1) to give the compound **II** (6.43 g, 83%) as a yellow oil. ^1H NMR (500 MHz, $CDCl_3$) δ 4.13 (t, *J* = 6.1 Hz, 2H), 3.22 (t, *J* = 6.8 Hz, 2H), 2.14 (quint, *J* = 6.6 Hz, 2H), 2.06 (s, 3H). ^{13}C NMR (126 MHz, $CDCl_3$) δ 171.0 (C), 64.2 (CH_2), 32.5 (CH_2), 21.0 (CH_3), 1.5 (CH_2). HRMS (ESI): *m/z* $[M+Na]^+$ calcd for $C_5H_9O_2INa$: 250.9539; found: 250.9535.

1.3 Synthesis of compounds 1 and 2

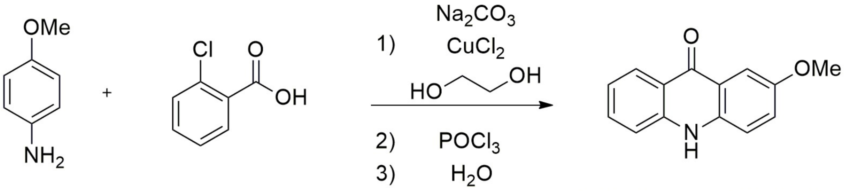

2-Methoxy-9(10H)-Acridone

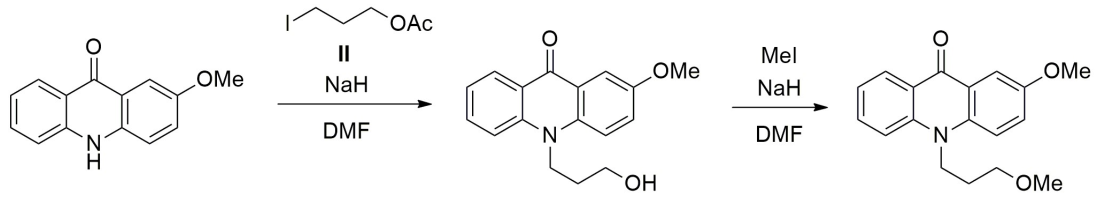

2-Methoxy-9(10H)-Acridone **1** **2**

2-methoxy-9-(10H)-acridone. This compound was prepared from 2-chlorobezoic acid and 4-methoxyaniline according to a previously described procedure (Smith et al., 2004). ^1H and ^{13}C NMR spectra matched to those of the reported one (Pintér and Klussmann, 2012).

Compound 1 (N-(3-hydroxypropyl)-2-methoxy-9-acridone). To a solution of 2-methoxy-9-(10H)-acridone (338 mg, 1.50 mmol) in anhydrous DMF (8 mL) at 70 ºC, NaH (60% in mineral oil, 180 mg, 4.50 mmol) was added. The mixture was stirred for 10-15 minutes and then compound **II** (684 mg, 3.00 mmol) was added. The reaction mixture was stirred at 70 ºC for 24 h. After this time, the reaction was allowed to cool to room temperature and the same amounts of both compound **II** and NaH was added. The reaction mixture was stirred at 70º C for another 24 h. The reaction was allowed to cool to room temperature and quenched by dropwise addition of water. The mixture was diluted with EtOAc and washed with HCl 10% (×5), the organic layer was separated and dried with anhydrous Na_2SO_4 and the solvent was removed. The residue was purified by flash chromatography (SiO_2 Hexane/EtOAc 4:6). In these reaction conditions, acetate group was hydrolyzed to give compound **1** (185 mg, 44%, 88% based on recovered starting material) as a yellow-brown oil. Recrystallization in hexane/dichloromethane mixtures gave compound **1** as green-yellow solid. ^1H NMR (600 MHz, MeOD) δ 8.40 (dd, *J* = 7.9, 1.3 Hz, 1H), 7.79 (d, *J* = 3.1 Hz, 1H), 7.76 – 7.75 (m, 3H), 7.43 (dd, *J* = 9.4, 3.1 Hz, 1H), 7.30 (ddd, *J* = 7.9, 6.4, 1.3 Hz, 1H), 4.62 – 4.55 (m, 2H), 3.90 (s, 3H), 3.78 (t, *J* = 5.7 Hz, 2H), 3.31 (br s, 1H), 2.11 – 2.04 (m, 2H). ^{13}C NMR (151 MHz, MeOD) δ 179.1 (C), 156.1 (C), 142.7 (C), 138.1 (C), 135.3 (CH), 128.1 (CH), 126.2 (CH), 123.7 (C), 122.34 (CH), 122.28 (C), 118.6 (CH), 116.6 (CH), 106.9 (CH), 59.9 (CH_2), 56.1 (CH_3), 44.4 (CH_2), 31.4 (CH_2). HRMS (ESI): *m/z* $[M+Na]^+$ calcd for $C_{17}H_{17}NO_3Na$: 306.1100 found: 306.1102.

<u>Compound 2 (N-(3-methoxypropyl)-2-methoxy-9-acridone).</u> To a solution of compound **1** (27 mg, 0.09 mmol) in anhydrous DMF (3 mL), NaH (60% in mineral oil, 23 mg, 0.57 mmol) was added. The resulting mixture was stirred for 10-15 minutes and then compound MeI (16 mg, 0.11 mmol) was added. The reaction mixture was stirred for another 2,5 h. After this time, the reaction was quenched by dropwise addition of water. The mixture was diluted with EtOAc and washed with HCl 10% (×5), the organic layer was separated and dried with anhydrous Na_2SO_4 and the solvent was removed. The residue was purified by flash chromatography (SiO_2, Hexane/EtOAc 7:3) to give compound **2** (27.2 mg, 96%) as a green-yellow oil. ^{1}H NMR (500 MHz, MeOD) δ 8.36 (d, J = 8.0 Hz, 1H), 7.73 (d, J = 2.6 Hz, 1H), 7.70 (d, J = 7.0 Hz, 1H), 7.68-7.62 (m, 2H), 7.34 (dd, J = 9.4, 2.6 Hz, 1H), 7.24 (t, J = 7.2 Hz, 1H), 4.44 (m, 2H), 3.86 (s, 3H), 3.47 (t, J = 5.4 Hz, 2H), 3.37 (s, 3H), 2.07 – 1.99 (m, 2H). ^{13}C NMR (126 MHz, MeOD) δ 178.9 (C), 156.0 (C), 142.6 (C), 138.0 (C), 135.2 (CH), 128.0 (CH), 126.1 (CH), 123.6 (C), 122.3 (CH), 122.2 (C), 118.5 (CH), 116.4 (CH), 106.9 (CH), 70.4 (CH_2), 59.1 (CH_3), 56.0 (CH_3), 44.3 (CH_2), 29.0 (CH_2). HRMS (ESI): m/z $[M+H]^+$ calcd for $C_{18}H_{20}NO_3$: 298.1443 found: 298.1441.

1.4 ^{1}H-NMR and ^{13}C-NMR spectra of new compounds

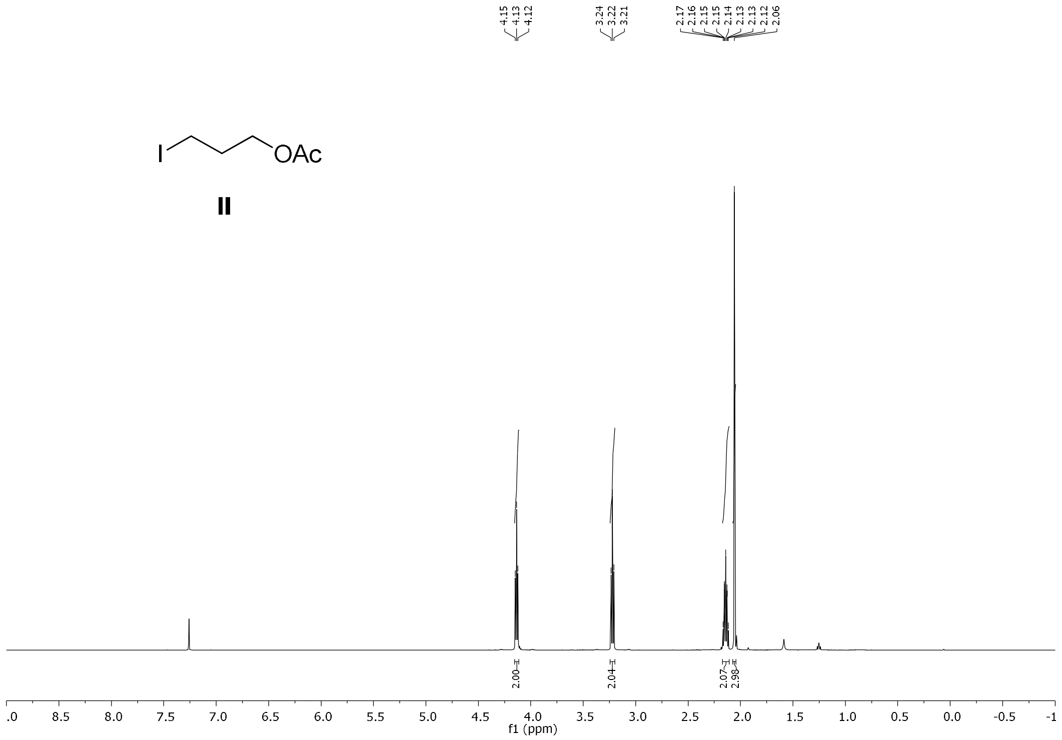

Supplementary Figure S1. ^{1}H NMR (500 MHz, $CDCl_3$) spectrum of compound **II**.

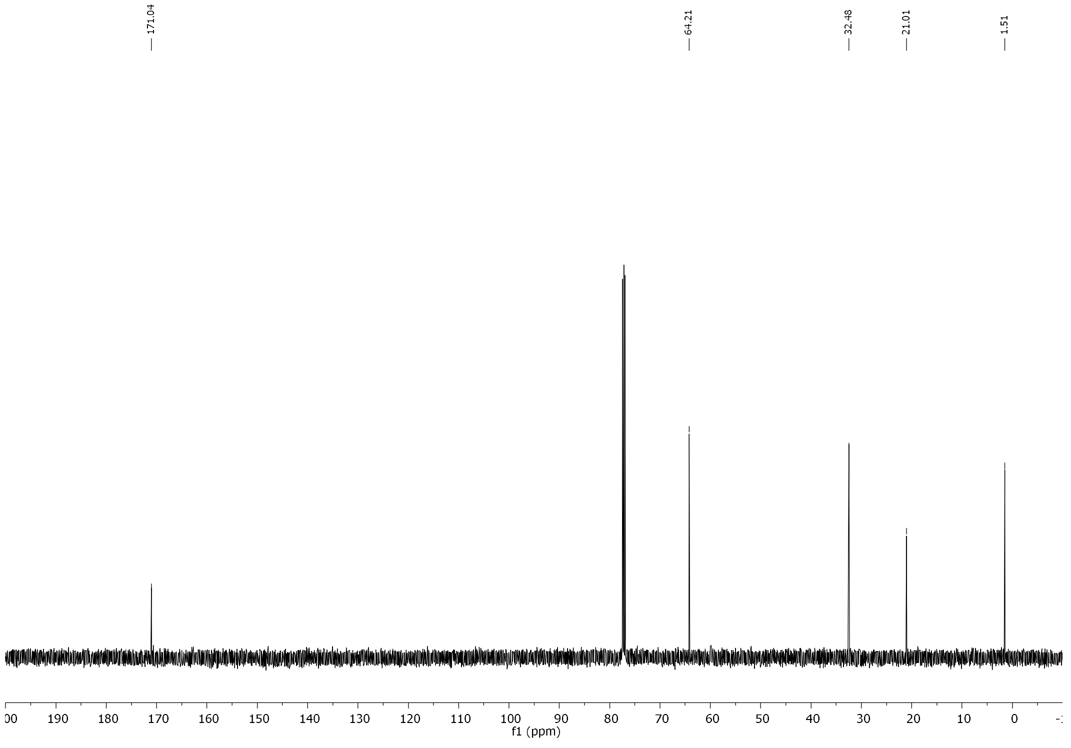

Supplementary Figure S2. ^{13}C NMR (126 MHz, $CDCl_3$) spectrum of compound **II**.

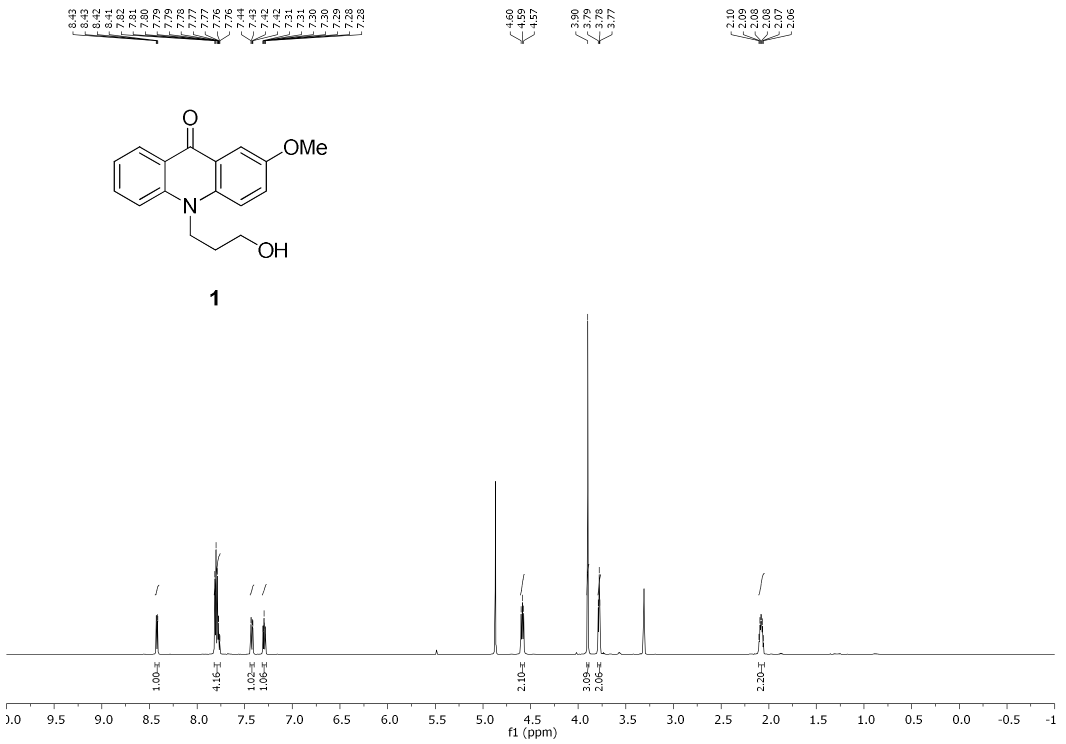

Supplementary Figure S3. ^{1}H NMR (600 MHz, MeOD) spectrum of compound **1**.

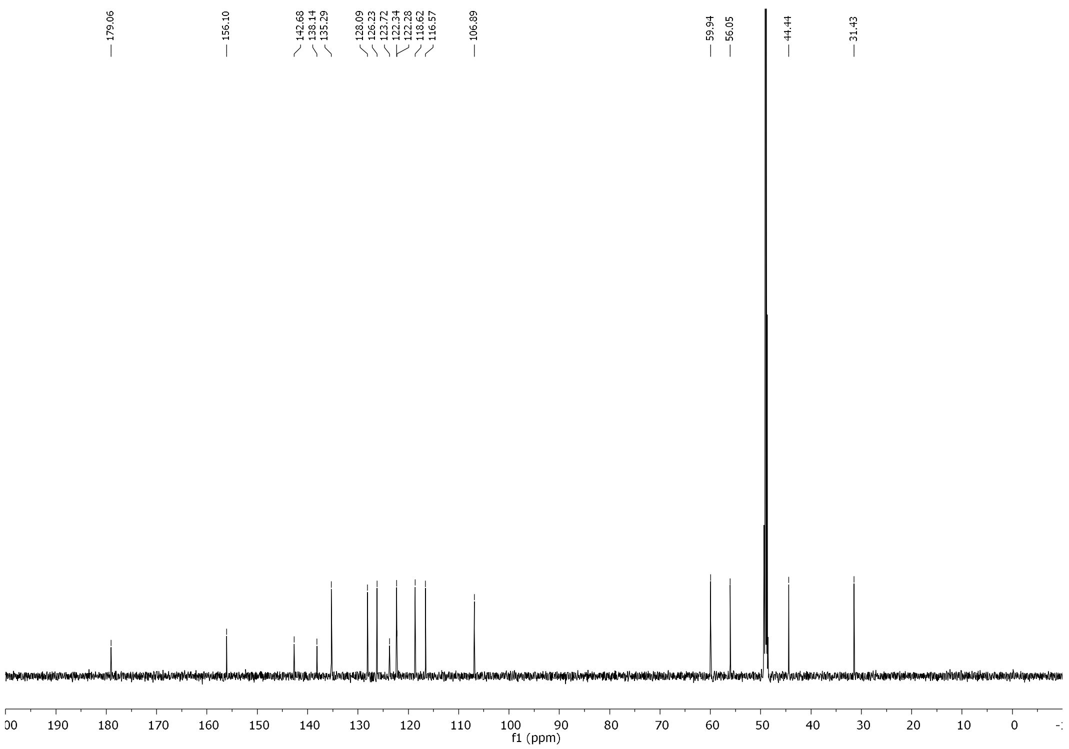

Supplementary Figure S4. ^{13}C NMR (151 MHz, MeOD) spectrum of compound **1**.

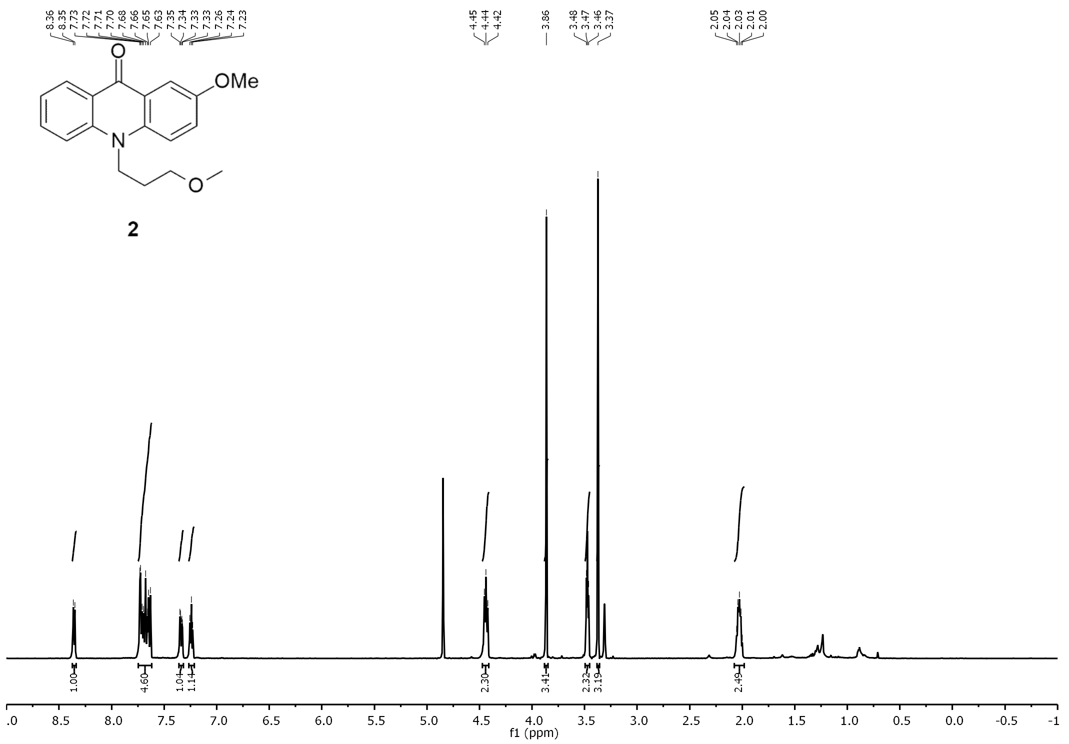

Supplementary Figure S5. ^{1}H NMR (500 MHz, MeOD) spectrum of compound **2**.

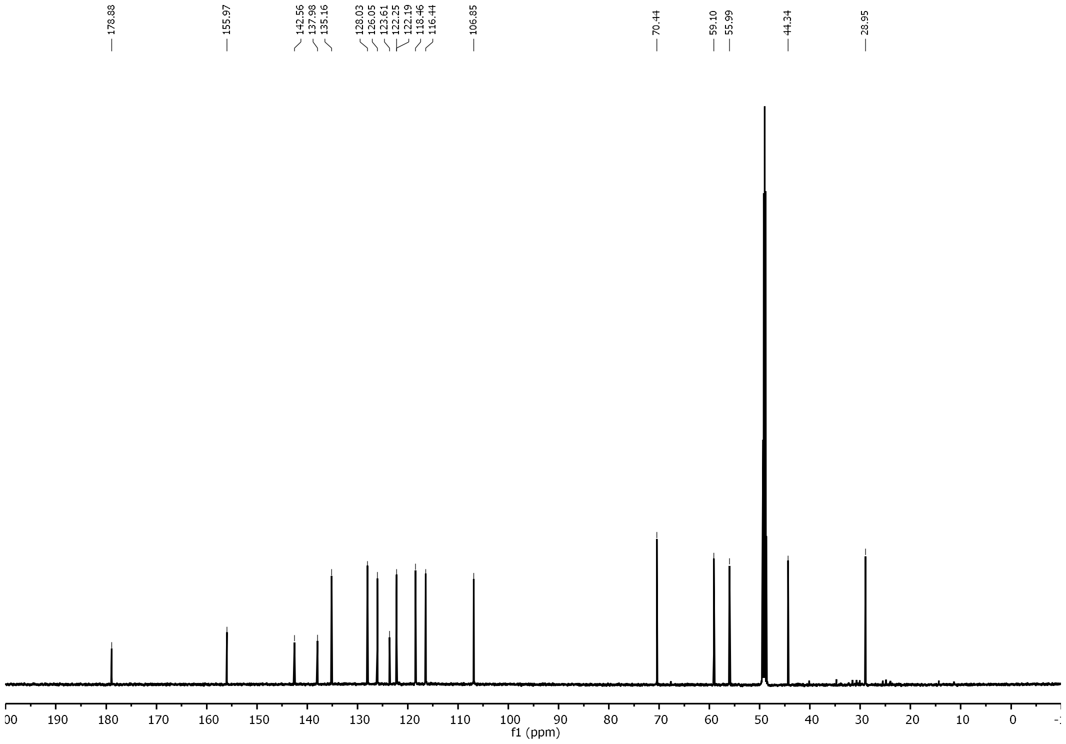

Supplementary Figure S6. ^{13}C NMR (126 MHz, MeOD) spectrum of compound **2**.

2 Supplementary Figures and Tables

2.1 Supplementary Figures

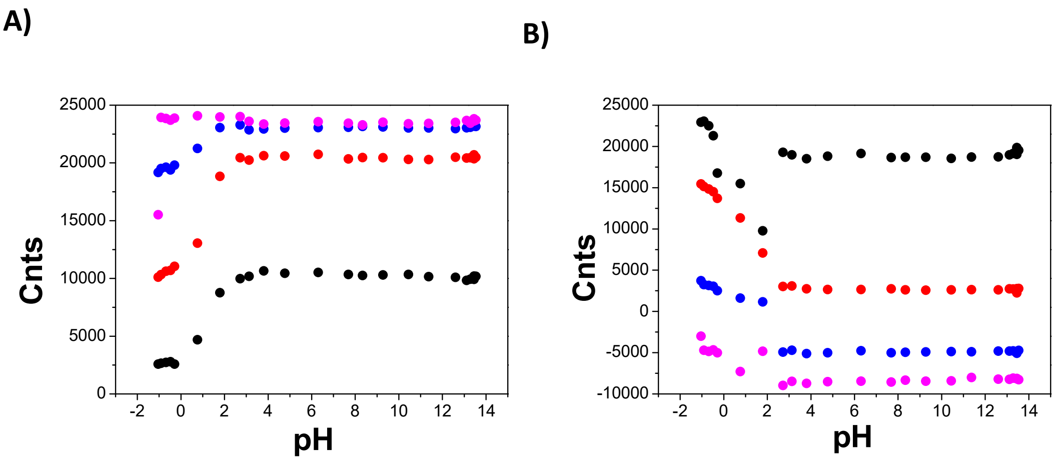

Supplementary Figure S7. Pre-exponential factors of the long (A) and the short (B) decay times of **1** as a function of pH at the emission wavelengths 440 (black), 470 (red), 500 (blue), and 530 nm (magenta).

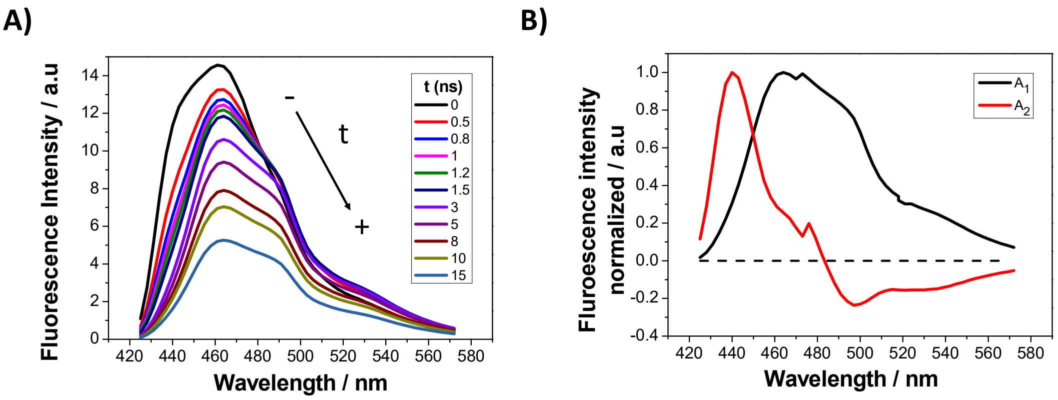

Supplementary Figure S8. TRES (A) and SAEMS (B) of **1** in aqueous solution at pH 6.15. The TRES spectra show the time evolution between 0 and 15 ns. The SAEMS show the emission spectrum associated to the longest (black) and the shortest decay time (red).

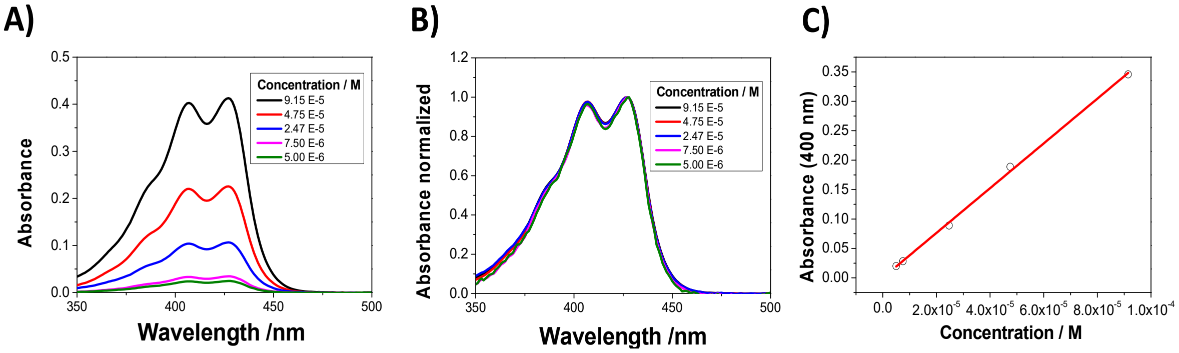

Supplementary Figure S9. Absolute (A) and normalized (B) absorption spectra of different concentrations of **1** in aqueous solution at pH 6.30. (C) Plot of the absorbance at 400 nm, from the spectra of panel (A), versus the concentration of **1**, showing an excellent linear relation, according to Beer's Law.

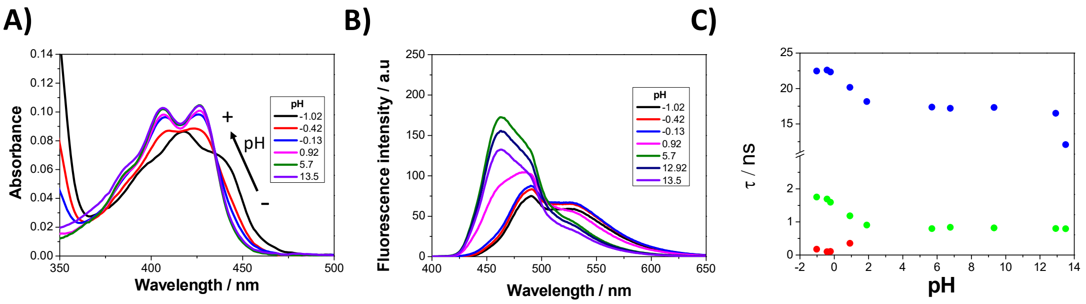

Supplementary Figure S10. (A) Absorption spectra, (B) steady-state emission spectra (λ_{ex} = 400 nm), and (C) fluorescence decay times of **2** in aqueous solution at different pH values.

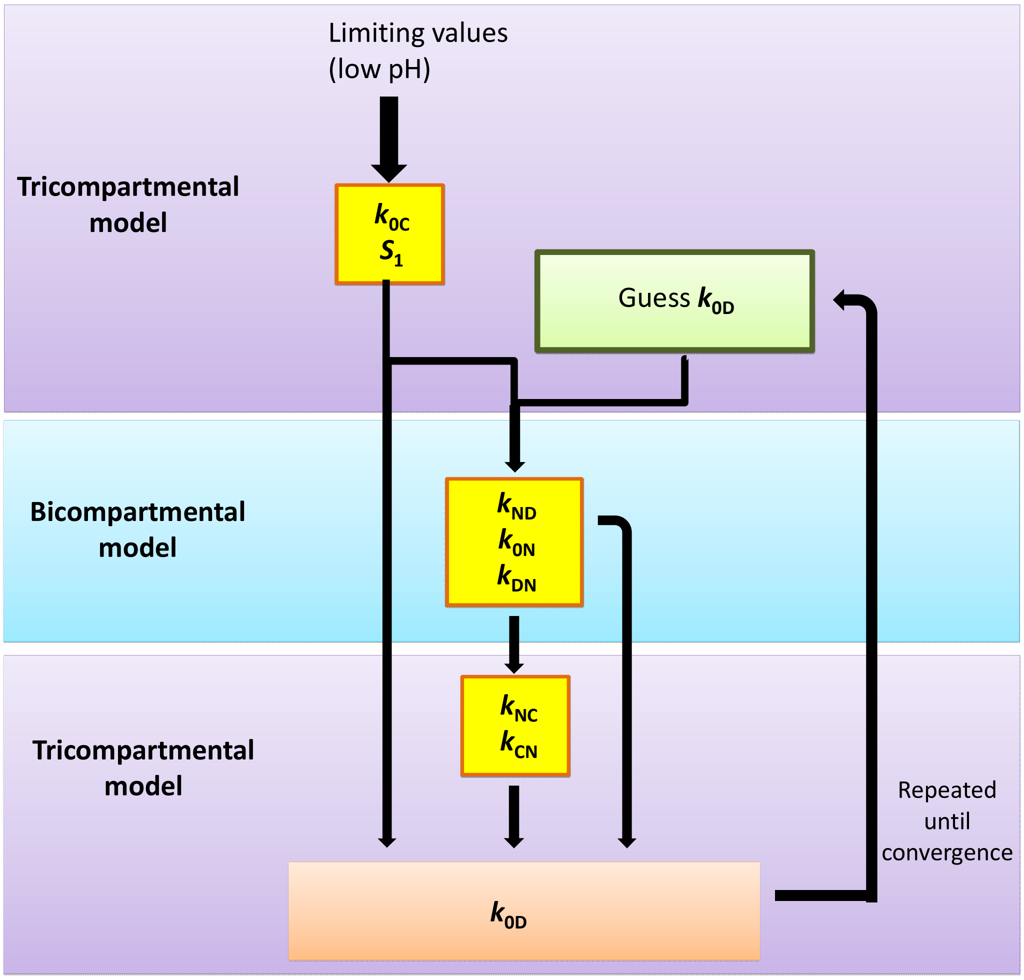

Supplementary Figure S11. Iterative fitting process to recover all the kinetic rate constants for the dynamic excited-state behavior of **1** in aqueous solution.

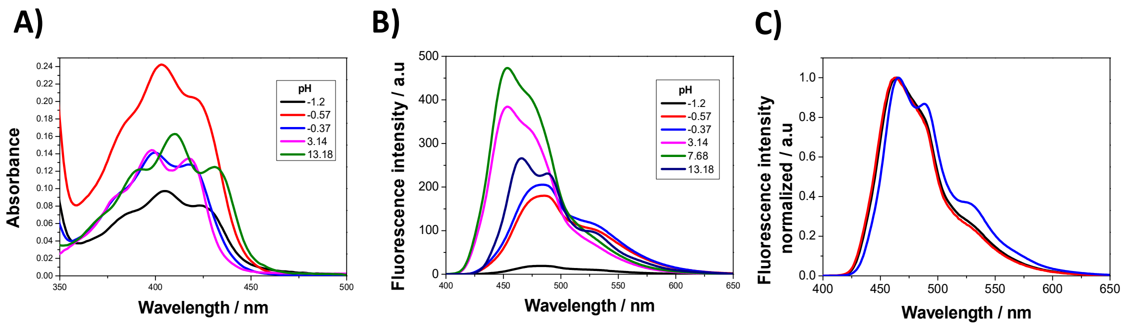

Supplementary Figure S12. (A) Absorption spectra and (B) steady-state emission spectra (λ_{ex} = 400 nm) of 2-methoxy-9(10H)-acridone in aqueous solution and different pH values. (C) Normalized emission spectra of **1** (black), **2** (red), and 2-methoxy-9(10H)-acridone (blue) at pH > 13.

2.2 Supplementary Tables

2.2.1 Table S1. DFT energies and number of imaginary frequencies of the studied forms in the gas phase.

Species	SCF Energy (au)	N. of Imaginary Frequencies
AN	–937.687715	0
AP	–938.027655	0
APC	–938.066261	0
Dim	–1875.397523	

2.2.2 DFT Geometries in the gas phase

2.2.2.1 Table S2. Representative bond distances and angles from the DFT-optimized geometries of the different studied species in the gas phase.

	Species			
**Bond Distances (Å)**	**AN**	**AP**	**APC**	**Dim**
C=O 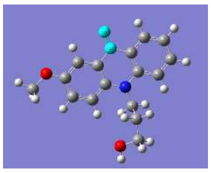	1.22564	1.21057	1.32278	1.23639<br>1.23731
C=O----H 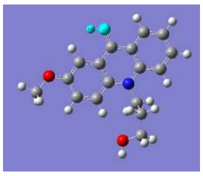	NA	NA	0.96640	1.83976<br>1.83072
N-C 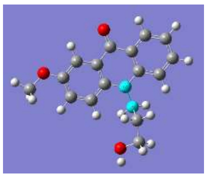	1.45756	1.53658	1.47577	1.46880<br>1.46813
N---H 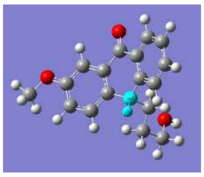	NA	1.02399	NA	NA

**Bond Angles (°)**	**AN**	**AP**	**APC**	**Dim**
C-N-C 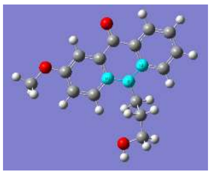	120.86026	113.80434	121.33077	120.57684<br>120.55915
**Dihedral Angle (°)**	**AN**	**AP**	**APC**	**Dim**
N-C-C-C 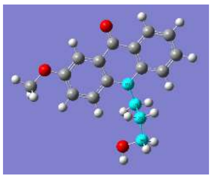	164.51677	-179.77162	155.12442	-69.07544<br>-61.91486
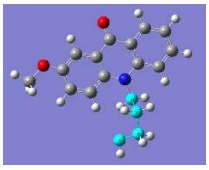 C-C-C-O	-62.49789	-56.45114	-59.91618	176.52459<br>177.51118
C-C-O-H 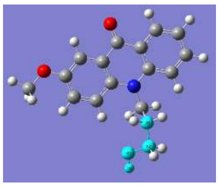	176.59529	-177.21425	178.89162	-145.80667<br>-61.00717

2.2.2.2 AN – atom coordinates

C	-4.26442900	-0.88644600	0.01368100
C	-3.24404800	0.03548200	-0.10944300
C	-1.89748900	-0.38192200	-0.13894000
C	-1.63369100	-1.76040900	-0.01438300
C	-2.68716400	-2.67306300	0.10804800
C	-3.99930200	-2.25431200	0.11867300
H	-5.28991200	-0.53073300	0.03593400
H	-3.49874800	1.08494300	-0.16322200
H	-2.41521700	-3.71921300	0.19433300
H	-4.81039300	-2.96719000	0.21486900
C	-0.25837100	-2.27561500	-0.00281000
C	0.79614100	-1.24963000	-0.04967900
C	0.47396100	0.11563800	-0.17189000
C	2.12287800	-1.66272700	0.04076900
C	1.53200600	1.03806300	-0.17115300
C	3.15786600	-0.74562500	0.02198500
H	2.32456800	-2.72361300	0.12830700
C	2.84779800	0.61384000	-0.07822500
H	1.34799100	2.10342600	-0.22041900
H	3.62975400	1.36293800	-0.07715500
N	-0.85827600	0.52577300	-0.28581800
O	-0.00476900	-3.47282700	0.06470400
C	-1.13970700	1.92877800	-0.56307000
H	-0.34462500	2.31834600	-1.19702300
H	-2.05077000	1.98910700	-1.16066500
C	-1.25721700	2.79415100	0.69361100
H	-2.19441700	2.58546100	1.21935000
H	-0.44346400	2.54487500	1.38100700
C	-1.17165800	4.27284800	0.36558300
H	-1.96674400	4.55171700	-0.34309900
H	-1.32034600	4.86578400	1.27831500
O	0.11164800	4.50541600	-0.19409400

H	0.17521300	5.42831500	-0.46210800
O	4.42196600	-1.24477000	0.11340000
C	5.49695400	-0.33394000	0.10299200
H	6.40451200	-0.93276000	0.17823700
H	5.52881400	0.24791500	-0.82637400
H	5.45378400	0.35706200	0.95385500

2.2.2.3 AP – atom coordinates

C	4.12854800	-1.22911100	-1.06940700
C	3.09675300	-0.33986800	-1.35164400
C	1.91599700	-0.44914000	-0.63889500
C	1.73356100	-1.40935300	0.34950700
C	2.78418200	-2.28031800	0.63021300
C	3.97606800	-2.18975200	-0.07381300
H	5.05460800	-1.16576000	-1.62936500
H	3.22333900	0.42154100	-2.11630100
H	2.63093900	-3.02864400	1.39972200
H	4.78760100	-2.87401400	0.14556900
C	0.42161600	-1.57755800	1.03355600
C	-0.72529800	-0.90232800	0.36466400
C	-0.51489100	0.04773900	-0.63714900
C	-2.01518900	-1.30988100	0.64685900
C	-1.56924100	0.55816400	-1.36788200
C	-3.09935600	-0.78866300	-0.07040300
H	-2.18299100	-2.05671900	1.41407100
C	-2.86637700	0.13566300	-1.09370800
H	-1.40426100	1.28602900	-2.15654600
H	-3.68322800	0.53240900	-1.68140700
N	0.85439300	0.56123800	-0.86096900
O	0.28560000	-2.28890400	2.00359100
C	1.16405700	1.80284800	-0.01032400
H	1.00130300	1.50887900	1.02577600

H	2.22808600	1.99530500	-0.15852100
C	0.32880700	3.01655800	-0.37143900
H	0.47940000	3.30386300	-1.41879800
H	-0.73214800	2.80986600	-0.21841200
C	0.72355200	4.18302600	0.52787400
H	1.76548900	4.47644400	0.33144300
H	0.08627500	5.04432800	0.29218100
O	0.54889300	3.73847600	1.85556800
H	0.74485400	4.45810200	2.46641000
O	-4.30453200	-1.24853200	0.28164200
C	-5.45670500	-0.78024500	-0.40835600
H	-6.30298700	-1.28533300	0.05246700
H	-5.57241300	0.30202200	-0.29297900
H	-5.41284000	-1.04029600	-1.47064900
H	0.91203800	0.84830000	-1.84220800

2.2.2.4 APC – atom coordinates

C	-4.33516000	-0.52883600	-0.01665600
C	-3.26415500	0.31759900	-0.13633400
C	-1.94543000	-0.19737700	-0.14612700
C	-1.77554000	-1.60038000	-0.00347400
C	-2.90968800	-2.44918900	0.10930100
C	-4.16837500	-1.92592100	0.09909400
H	-5.33526800	-0.10912700	-0.00420400
H	-3.44326800	1.38078800	-0.19698100
H	-2.74503200	-3.51449900	0.20830000
H	-5.03479500	-2.57010200	0.18820100
C	-0.47445900	-2.13085100	0.02240500
C	0.65294200	-1.27809500	-0.03326400
C	0.43083200	0.11687500	-0.17887200
C	1.96988300	-1.77824800	0.05605200

C	1.56061700	0.96860000	-0.19910300
C	3.05542100	-0.93704000	0.01312600
H	2.18273800	-2.83547500	0.17453600
C	2.82825400	0.45491600	-0.10636300
H	1.45040400	2.04450000	-0.25264200
H	3.66423900	1.14261800	-0.11460800
N	-0.85002200	0.61517000	-0.28711800
O	-0.37330500	-3.44621000	0.11914400
C	-1.02645400	2.05338300	-0.56696600
H	-0.21039100	2.36327900	-1.21469000
H	-1.93480000	2.17232600	-1.15406300
C	-1.04427600	2.91925500	0.69522300
H	-2.03394900	2.91874800	1.16092200
H	-0.34387600	2.51020300	1.42931200
C	-0.61884500	4.34293200	0.37680600
H	-1.30770000	4.79854600	-0.34872200
H	-0.64881800	4.94727500	1.29124100
O	0.69670800	4.26318800	-0.15017200
H	1.00402700	5.14614600	-0.38438800
O	4.26857000	-1.50270900	0.10364700
C	5.42908800	-0.68001100	0.07127200
H	6.27422100	-1.36083400	0.15023400
H	5.49781300	-0.12723100	-0.87071100
H	5.44643500	0.01575600	0.91583800
H	0.54444600	-3.74893200	0.11457800

2.2.2.5 Dim – atom coordinates

C	-1.16262300	-0.87592700	-2.58276300
C	0.03569100	-1.48420300	-2.25913800
C	1.17167200	-0.73204200	-1.91402300
C	1.03921800	0.67294800	-1.90968000
C	-0.17823400	1.27651400	-2.23634000

C	-1.28206500	0.51747200	-2.57026900
H	-2.00838900	-1.50499700	-2.82988000
H	0.06267100	-2.56354000	-2.26274100
H	-0.26001100	2.35675000	-2.20879800
C	2.18086800	1.52552700	-1.57642100
C	3.42219100	0.82722700	-1.25892800
C	3.49047800	-0.58209500	-1.24633800
C	4.54932600	1.59797000	-0.93708000
C	4.71876000	-1.16843500	-0.86878800
C	5.74369800	1.01019800	-0.59645200
H	4.42651900	2.67437300	-0.96879500
C	5.81085200	-0.38647200	-0.55917100
H	4.82485000	-2.24019900	-0.79498500
H	6.73665700	-0.87627100	-0.27420200
N	2.38253800	-1.34494200	-1.58798200
O	2.11229100	2.75977500	-1.55226100
C	2.45470200	-2.81101800	-1.61654300
H	1.94014300	-3.15005700	-2.51796200
H	3.49326500	-3.09925900	-1.76207700
C	1.86624700	-3.50149700	-0.38450400
H	0.80942400	-3.23857400	-0.27895200
H	1.90763900	-4.58452200	-0.54446900
C	2.55628700	-3.17196100	0.93782700
H	3.59245800	-3.52864300	0.92853000
H	2.59396300	-2.08105000	1.07404300
O	1.92132700	-3.80121500	2.02518400
H	1.00627100	-3.46358100	2.05325200
C	-4.54476000	-0.10749900	0.16874600
C	-3.70952200	0.84539000	0.72190500
C	-2.49322400	0.48917000	1.33092800
C	-2.14621600	-0.87611600	1.32107100
C	-2.99086800	-1.82816500	0.74837900
C	-4.19343500	-1.46261400	0.17795300

H	-5.47501300	0.22186400	-0.27740300
H	-4.02075300	1.87742600	0.65993800
H	-2.68221000	-2.86609700	0.76887000
C	-0.88815800	-1.33372100	1.91041500
C	-0.08461900	-0.30951800	2.56642400
C	-0.47961100	1.04779900	2.54568300
C	1.10205300	-0.69441900	3.21411500
C	0.37543300	1.98271300	3.16927300
C	1.90874100	0.23030800	3.83254500
H	1.36925300	-1.74516100	3.20306400
C	1.53293800	1.57652400	3.79671800
H	0.15208500	3.03845700	3.15326800
H	2.16225900	2.32741500	4.26375900
N	-1.66232700	1.43552800	1.92864500
O	-0.54981800	-2.52204400	1.84436400
C	-2.07288800	2.84511400	1.88524500
H	-3.14500400	2.88278700	2.08324100
H	-1.61707400	3.36554200	2.72468700
C	-1.74625500	3.54786000	0.56661300
H	-2.14460100	2.97462200	-0.27612000
H	-2.25195500	4.51986400	0.55485900
C	-0.25764700	3.76402300	0.33187900
H	0.15054500	4.41597400	1.12259800
H	0.27211600	2.80668100	0.40460500
O	-0.07255500	4.34161700	-0.93803000
H	0.76413400	3.98289600	-1.28547600
H	6.61153600	1.61152500	-0.35089300
O	-2.42819900	1.19491000	-2.85838500
C	-3.51881900	0.46136700	-3.36905400
H	-3.90476800	-0.25655700	-2.63616900
H	-4.29590600	1.19150900	-3.59615500
H	-3.25051100	-0.07295700	-4.28848400
H	2.82200800	-0.07587200	4.32935800

O	-4.95312600	-2.45944000	-0.35688100
C	-6.23900700	-2.13266400	-0.83491400
H	-6.19671500	-1.44834700	-1.69159800
H	-6.69206900	-3.07046300	-1.15561200
H	-6.86086400	-1.68301100	-0.05175100

3 References

Chen, C., and Hong, S.H. (2012). Selective catalytic sp3 C-O bond cleavage with C-N bond formation in 3-alkoxy-1-propanols. *Org Lett* 14**,** 2992-2995.

Pintér, Á., and Klussmann, M. (2012). Sulfonic Acid-Catalyzed Autoxidative Carbon-Carbon Coupling Reaction under Elevated Partial Pressure of Oxygen. *Advanced Synthesis & Catalysis* 354**,** 701-711.

Smith, J.A., West, R.M., and Allen, M. (2004). Acridones and quinacridones: Novel fluorophores for fluorescence lifetime studies. *J. Fluoresc.* 14**,** 151-171.
